# A cancer persistent DNA repair circuit driven by MDM2, MDM4 (MDMX), and mutant p53 for recruitment of MDC1 and 53BP1 on chromatin

**DOI:** 10.1093/nar/gkaf627

**Published:** 2025-07-08

**Authors:** Viola Ellison, Alla Polotskaia, Gu Xiao, Pamella Leybengrub, Rusia Lee, Weigang Qiu, Ronald C Hendrickson, Wenwei Hu, Jill Bargonetti

**Affiliations:** Department of Biological Sciences, Hunter College City University of New York, Belfer Research Building, New York, NY 10021, United States; Department of Biological Sciences, Hunter College City University of New York, Belfer Research Building, New York, NY 10021, United States; Department of Biological Sciences, Hunter College City University of New York, Belfer Research Building, New York, NY 10021, United States; Department of Biological Sciences, Hunter College City University of New York, Belfer Research Building, New York, NY 10021, United States; Department of Biological Sciences, Hunter College City University of New York, Belfer Research Building, New York, NY 10021, United States; Department of Biology and Biochemistry, The Graduate Center City University of New York, NY 10016, United States; Department of Biological Sciences, Hunter College City University of New York, Belfer Research Building, New York, NY 10021, United States; Department of Biology and Biochemistry, The Graduate Center City University of New York, NY 10016, United States; Department of Biochemistry and Molecular Biology, Sylvester Comprehensive Cancer Center, University of Miami, Miami, FL 33136, United States; Department of Radiation Oncology, Rutgers Cancer Institute of New Jersey, Rutgers University, New Brunswick, NJ 08901, United States; Department of Biological Sciences, Hunter College City University of New York, Belfer Research Building, New York, NY 10021, United States; Department of Biology and Biochemistry, The Graduate Center City University of New York, NY 10016, United States; Department of Cell and Developmental Biology, Weill Cornell Medical College, NY 10065, United States

## Abstract

DNA damage signaling requires functional interactions between 53BP1 and the wild-type p53 tumor suppressor. Cancer cells often express elevated levels of transcriptionally inactive mutant p53 (mtp53) that maintains MDM2 and MDMX (MDM4) binding partners. The ability of mtp53 to functionally interact with additional proteins in the context of a dynamic equilibrium with MDM2–MDMX heterodimers has not been described. Employing a stable isotope labeling in cell culture analysis in T47D breast cancer cells (expressing mtp53 L194F), we uncovered several chromatin-associated DNA replication and repair factors as MDM2-regulated phosphoproteins, including 53BP1. We used proximity ligation analysis in multiple breast cancer cell lines to confirm 53BP1–MDM2 complex formation. We demonstrated that a mtp53–MDM2/MDMX complex promoted 53BP1–MDC1 interactions by showing that mtp53–MDM2/MDMX complex disruptors, Nutlin 3a and ALRN-6924, reduced the 53BP1–MDC1 nuclear interactions (especially in S-phase). Surprisingly, these MDM2-driven MDC1–53BP1 interactions were not ATM dependent, suggesting distinct 53BP1–MDC1 complexes in response to genotoxic stress. We found that MDM2-deficient cells have increased poly-ADP-ribosylation on chromatin which supports the possibility that a mtp53–MDM2/MDMX pathway promotes aberrant DNA repair. Taken together, our data suggest that a mtp53–MDM2/MDMX complex orchestrates DNA repair machinery activity on chromatin, thus priming cancer cells for persistent DNA damage repair (CPR).

## Graphical Abstract

**Graphical Abstract:**
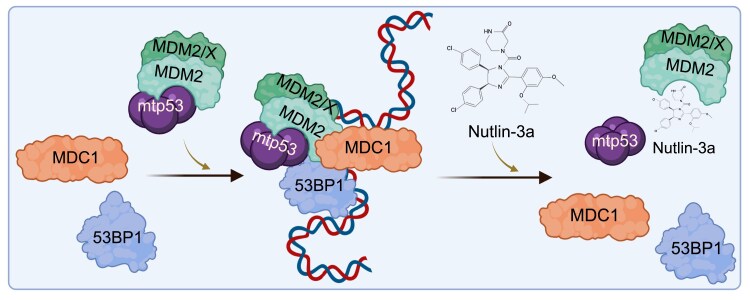

## Introduction

The normal *TP53* gene encodes the site-specific DNA binding transcription factor p53, which is an evolutionarily conserved multifunctional genome guardian that is the most highly mutated gene in cancers [1]. The wild-type p53 (wtp53) protein functions as a transcription factor to promote genome maintenance through a combination of transcription activation of target genes, direct DNA damage recognition, and complex regulation of DNA repair activities [2–4]. While wtp53 is conserved from*Caenorhabditis elegans* to human, its negative regulator and primary transcriptional target gene, MDM2, is not; there is no identified *MDM2* homolog in either *Drosophila or C. elegans* [5, 6]. In cells that have MDM2, wtp53 and MDM2 crosstalk to regulate when wtp53 can induce expression of hundreds of genes that collectively determine one of three broad cell fate outcomes, which are cell cycle arrest, senescence, or cell death [1]. MDM2 in a complex with its paralog MDMX (also called MDM4) constitutes an E3 ubiquitin ligase complex that targets wtp53 for ubiquitin-mediated proteolysis in un-genotoxic stressed conditions [1]. MDM2 and MDMX are RING domain E3 family members that function as dimers and contain N- terminal and C-terminal regions that bind and down-regulate wtp53 and many other proteins [7]. Although MDM2 (but not MDMX) homodimers are active, the specific activity of the MDM2/MDMX heterodimer is significantly higher due to MDMX-mediated E2 recruitment [8]. Both MDM2 and MDMX have been shown to interact with proteins that are part of DNA repair pathways [9]. While wtp53 is well known for its interaction with the DNA repair protein 53BP1, only a few studies have examined the interaction of mutant p53 (mtp53) with 53BP1 and none in the context of changes to MDM2 and MDMX in cancer cells [10–14].

In many cancers, and in particular breast cancer, the *TP53* gene contains missense mutations and the *MDM2* gene is over expressed [15]. The proteins produced by these altered alleles promote aggressive cancer and metastasis [16, 17]. In the case of missense mutant p53 (mtp53) proteins, many reported gain-of-function (GOF) properties are a result of their altered DNA-binding activity, and their ability to participate in novel protein–protein interactions in addition to those defined by wtp53 [18–21]. Examples of mtp53 GOF properties that influence DNA metabolism to promote cell survival have been reported by many laboratories and include modulation of poly (ADP-ribose) polymerase 1 (PARP1) processing of DNA replication and repair intermediates [22–24], and Treslin function during replication fork assembly [25].

A conspicuous property of GOF mtp53 in cancer cells is that it is maintained at high levels in the cell despite its ability to form complexes with MDM2–MDMX [26–28]. These observations suggest mtp53 may work in concert with MDM2 and MDMX to promote tumorigenesis [29, 30]. Like mtp53, MDM2 has been shown to regulate DNA metabolism by modulating histone post-translational modifications and chromatin-based functions of several proteins including PARP1 [31]. Moreover, MDM2 splice variants, also found over-expressed in breast cancer, have been shown to promote mtp53 GOF activities [30, 32].

To identify MDM2–MDMX chromatin-associated protein signatures that drive breast cancer, we conducted a SILAC phospho-proteome screen in MDM2-proficient and deficient T47D breast cancer cells (mtp53 L194F). We found reduced recovery of several proteins involved in shaping the nuclear landscape, including the DNA damage response (DDR) highlighted by inclusion of the DNA repair proteins p53 binding protein 1 (53BP1) and mediator of dna damage checkpoint 1 (MDC1). Using a combination of techniques and the p53-MDM2 disruptors Nutlin 3a (herein referred to as N3a) and ALRN-6924, a hierarchy of protein–protein interactions including MDM2–53BP1 and, surprisingly, 53BP1–MDC1 were revealed as mtp53–MDM2–MDMX dependent. Interestingly, we found that MDM2–driven 53BP1–MDC1 interactions did not require ATM activity, and the loss of 53BP1–MDC interactions in MDM2-deficient cells correlated with increased chromatin-associated PARylation. This suggested the removal of MDM2, and the loss of 53BP–MDC interactions increased replication stress. We propose that the pre-assembly of MDC1–53BP1 by MDM2–MDMX working in a complex with mtp53 functions to prime cancer persistent repair (CPR) to keep tumor cells viable. Through this novel GOF of mtp53, 53BP1 may promote error prone repair and thus contribute to mtp53-dependent genomic instability and metastasis [33].

## Materials and methods

### Chemicals and antibodies

Solvents and standard chemicals for buffers were obtained from Sigma–Aldrich (St. Louis, MO, USA) and Fisher Scientific unless otherwise indicated. Antibodies used for western blotting (WB), immunofluorescence staining (IF), immunoprecipitation (IP), and proximity ligation assay (PLA) were purchased from the following (usage denoted in parenthesis): rabbit p53 Sigma cat# A300-247A (PLA), and Proteintech cat# 10442-1- AP (WB); mouse p53 DO1 Santa Cruz Biotechnology cat# sc-126 (PLA and WB); mouse p53 DO1-HRP Santa Cruz Biotechnology cat# sc-126 HRP (WB); rabbit MDMX Proteintech cat# 17914-1-AP (WB); [5] rabbit MDM2 R&D Systems cat# AF1244 (WB); rabbit 53BP1 Cell Signaling Technology cat# 4937 (WB and IF); rabbit phospho-Serine 177853BP1 Cell Signaling Technology cat# 2675 (WB and IF); rabbit phospho-Serine 2553BP1 Sigma cat# PLA 0126 (WB, IF, and PLA); rabbit MDC1 Sigma cat# PLA0016 (WB, IF, and PLA); rabbit MCM4 Cell Signaling Technology cat# 12973 (WB); mouse Actin-HRP Sigma cat# A3854 (WB); [12] mouse Lamin A cat# SAB4200420 (WB); mouse PARP1 BD Biosciences cat# 51-6639GR (WB); goat 53BP1 Sigma cat# PLA0303 (PLA and IP); goat anti-mouse HRP Sigma cat# A3682 (WB); goat anti-rabbit Proteintech cat# SA00001-2 (WB); mouse Cyclin A Santa Cruz Biotechnology cat# sc-271682 (WB); rabbit Cyclin A Cell Signaling Technology cat# 67955S (IF); mouse Cyclin B Santa Cruz Biotechnology cat# sc-245 (WB); rabbit p21 Cell Signaling Technology cat# 2947S (WB); γH2AX phospho-Ser139 Cell Signaling Technology cat# 9718S (WB and IF); rabbit Poly ADP-Ribose Cell Signaling Technology cat# 83732S (WB); mouse MDM2 SMP14 Santa Cruz Biotechnology cat# sc-965 (IP); mouse IgG Santa Cruz Biotechnology cat# sc-2025 (IP); Purified mouse MDM2 4B2 [34]; and purified mouse MDM2 2A9 [34] were used for PLA and IP and prepared as described [35]. ALRN-6924-DP was a gift from Aileron Therapeutics. Nutlin3a (cat# S8059), Talazoparib (cat# S7048), and Temozolomide (cat# S-1237) were purchased from Selleckchem, Cathepsin L inhibitor Z-FY-CHO from MedChemExpress (cat# HY-128140), Etoposide (cat# E1383) and Benzonase (cat# 70746-3) from Sigma, and Aphidicolin from ApexBio (B7832). Stock solutions for drugs were prepared in 100% sterile DMSO (G-Biosciences cat# 786-1388).

### Cell culture and drug treatments

All cell lines, MDA-MB-468 (ATCC), MDA-MB-468 CRISPR clone G6 (R273H*fs*347Δ360–393) [36], MDA-MB-231 (ATCC), MCF7 (ATCC), T47D mlp [37], T47D mlp*shmdm2* [37], T47D.mlp*shmdmx* [37], were cultured in complete media (Dulbecco’s Modified Eagle Medium with 4.5 g/l glucose containing 10% fetal bovine serum, 50 μg/ml penicillin/streptomycin) at 37°C with 5% CO_2_ and passaged by trypsinization and dilution. For all drug treatments, culture medium on cell populations was removed and fresh medium containing either the working concentration of agent or vehicle (DMSO or H_2_O) was added, followed by incubation of cells for the indicated time.

### Phosphoproteome SILAC screen of chromatin targets

We carried out Stable Isotope Labeling of Cell Culture SILAC using T47D cells with or without MDM2 expression. We fractionated the T47D.*shmdm2 cell* extract (with and without MDM2 knockdown) into chromatin, nuclear soluble, and cytoplasmic fractions [38]. We isolated phospho-peptides before mass spectrometry by their strong affinity for metal ions on strong cation exchange (SCX) chromatography, which separates peptides by solution charge followed by immobilized metal affinity chromatography (IMAC) to enrich the phospho-peptides [39, 40]. This was followed by two-dimensional (2D) nano-liquid chromatography–mass spectrometry (LC/MS). Data are available via ProteomeXchange with identifier PXD061454. We compared the outcome chromatin-associated phospho-peptides and identified numerous phospho-peptides were involved in an MDM2 signal transduction cascade that influenced the chromatin. A total of *N* = 1381 phospho-peptides were identified from SILAC experiments. The fold change of intensities (examined as the heavy to light ratio) of these peptides between the samples with and without MDM2 knockdown (*y*-axis) were plotted against their average intensities (*x*-axis, log2 scale). Our customized interactive plot showing all peptides is available at https://borreliabase.org/∼wgqiu/clickme-khalikuz/temp-Points.html

### Flow cytometry

Cultures were harvested by trypsinization, collected by centrifugation (500 × *g*), washed with ice-cold Phosphate Buffered Saline (PBS) twice, and then resuspended in ice-cold PBS at a density of 1 × 10^6^ cells/ml. Cells were fixed in a final concentration of 70% Ethanol by adding the cell suspension dropwise into a solution of 100% ethanol while vortexing continuously. Fixed cells were stored at −20°C for 24 h, and then collected by centrifugation, washed twice with ice-cold PBS, and then resuspended and incubated at 37°C for 15 min in 500 μl of propidium iodide staining solution (0.1% Triton X-100, 200 μg/ml RNAse A, and 60 μM propidium iodide in PBS). Following staining, cell samples were filtered through a nylon mesh into polystyrene tubes, and then analyzed on a BD™ FACSCalibur instrument. Minimally, 10 000 events were counted for each sample and acquired data were analyzed using BD™ CellQuest or FlowJo software.

### Immunofluorescence staining

Cells were cultured on 12-well uncoated plates (MatTek, Cat# P12G-1.5-14-F) until ∼60% confluency, washed 3× with cold PBS (1 min/wash), and then fixed at room temperature (r.t.) with 4% paraformaldehyde in PBS for 15 min. Post-fixation, cells were washed 3× with 1 ml of cold PBS (5 min/wash), permeabilized at r.t. for 15 min with cold IF Permeabilization Buffer (0.5% Triton X-100 in PBS), blocked at r.t. for 1 h with IF Blocking Buffer (5% Normal Goat Serum, 0.2% Triton X-100 in PBS), and then incubated at r.t. for 2 h with primary antibodies (1:1000 53BP1; 1:3000 53BP1^ser25^; 1:1000 53BP1^ser1778^; 1:1000 Cyclin A2). After incubation with primary antibodies, cells were washed 3× with cold PBS, and then incubated at r.t. for 1 h with the secondary antibody in the dark (anti-rabbit Alexa Fluor 594; ThermoFisher Cat #11012). After washing with PBS as described above, cells were stained for 5 min with 10 μM Hoescht 33342 in PBS, washed at r.t. 1 min with PBS, and then allowed to dry overnight at 4°C before mounting using CitiFluor AF1 (50 μl/well; cat# 17970-25). Images were taken using the Nikon A1 confocal microscope and processed with the Nikon NIS Element software, ImageJ, and Cell Profiler.

### EdU labeling for measurement of S-phase population

For measurement of the S-phase population, cells grown in 12-well glass bottom plates were incubated with 20 μM EdU added directly to the culture medium (Invitrogen; cat# A10044) for 15 min. Following fixation and permeabilization as specified in the immunofluorescence protocol, cells were washed twice with ice-cold 3% BSA in PBS for 5 min, once with PBS for 5 min, and then incubated in the dark at r.t. for 30 min with either Biotin-Azide (APExBio; cat# A8013), Alexa Fluor 594 or 647-Azide (Invitrogen; cat# A10270 and A10277, respectively) under click chemistry reaction conditions [100 μl/well 4 μM Azide compound, 1 mM copper sulfate (Sigma; cat# 451657), 5 mM sodium ascorbate (freshly prepared, Sigma; cat# A7631), 50 mM Tris, 150 mM NaCl, final pH 7.5]. After the incubation, the reaction buffer was discarded and cells were washed (1 ml/well, 5 min/wash) once with PBS, once with 3% BSA in PBS, once with PBS, and then blocked and subjected to IF staining for specific antigens; all steps were performed in the dark.

### Proximity ligation assay

The protocol for the PLA was performed as described previously [24] using the Sigma Aldrich Duolink System™ [anti-mouse, rabbit or goat (-) or (+) secondary antibodies; either 594 (cat# DUO92008) or 647 (cat# DUO92013) detection kit] with modifications indicated below (unless indicated otherwise, all incubations were performed as specified by the manufacturer). Cells were seeded in 12-well un-coated glass-bottom plates (MatTek) at 1 × 10^5^ cells per well in complete media, cultured until 50% confluency, and then washed 3× with ice-cold PBS for 1 min/wash followed by fixation with 4% paraformaldehyde in PBS for 15 min. Next, cells were permeabilized with ice-cold PBS containing 0.5% Triton X-100 for 15 min, blocked, and then incubated with primary antibodies (55 μl/well) for 2 h using the indicated dilutions (prepared in Duolink Ab diluent): goat anti-53BP1 1:1000; rabbit anti-p53 Sigma 1:2000; rabbit anti-MDC1 1:2000; mouse anti-MDM2 4B2 1:500 (final concentration: 1 μg/ml) or 2A9 1:1000 (final concentration: 1 μg/ml); mouse anti-Biotin 1:1000. After washing 3× (1 ml/wash), cells were incubated with the appropriate PLA Plus/Minus pair of secondary antibody probes (55 μl/well), and then subjected to the ligation and amplification steps. After the amplification step cells were washed 2× with Buffer B as indicated, 1× with 0.01× Buffer B containing 1 μg/ml Hoescht 333243 for 5 min, once with 0.01× Buffer B for 1 min, and then air dried in the dark at 4°C after first removing remaining wash buffer by pipetting. Cells were mounted as described in the immunofluorescence protocol, and images were taken using the Nikon A1 confocal microscope and processed with the Nikon NIS Elements, Cell Profiler, and ImageJ software.

### Cell extract preparation, SDS–PAGE, and western blot analysis

Total cell lysates were prepared using Phosphate/Chaps (PC) Buffer (50 mM KPO_4_, 1 mM Dithiothreitol (DTT), 1 mM Ethylenediaminetetraacetic acid (EDTA), 50 mM NaF, 50 μM NaV, and 10 mM β-glycerolphosphate, 7 mM CHAPS detergent (3-((3-cholamidopropyl) dimethylammonio)-1-propanesulfonate), 10% glycerol, 350 mM NaCl, pH 7.4 supplemented with PierceComplete™ protease inhibitor cocktail tablet (ThermoFisher Cat# A32961) as specified by the manufacturer. Sub-confluent cell cultures from 6 cm plates were harvested by either trypsinization or scraping using a rubber policeman, collected by low-speed centrifugation, washed by resuspending in 5 ml cold PBS, re-pelleted, and then resuspended in ice-cold PC Buffer (1 × 10^6^ cells/100 μl buffer). Resuspended cells were incubated on ice for 30 min, and cell lysates were clarified by centrifugation at 10 000 rpm at 4°C for 30 min in an Eppendorf 5415R microfuge. After determining the protein concentration of each sample using the Bio-Rad Bradford assay, aliquots (10–25 μg) of cell lysates were prepared for SDS–PAGE by mixing with an equal volume of 2× NuPAGE™ sample buffer containing 50 mM DTT, heated at 75°C for 10 min, and then incubated at r.t. for 10 min after addition of iodoacetamide to a final concentration of 50 mM to inhibit disulfide bond formation. Samples were then loaded onto an appropriate concentration SDS–PAGE gel (typically 8% gels; 30% acrylamide:0.4% Bis-acrylamide) for western blot analysis (electroblotting performed onto nitrocellulose membrane). Blots were blocked using 5% Non-Fat Dry Milk Buffer (NFDM Buffer; 5% NFDM in 10 mM Tris pH 7.4, 150 mM NaCl), and all primary antibodies were diluted 1:1000 in 1% NFDM Buffer containing 0.1% Triton X-100 except for the following: rabbit p53 Proteintech was used at 1:10 000–1:20 000; rabbit MDM2 R&D Systems were diluted in 3% BSA in PBS containing 0.1% Triton X-100. Primary antibodies were detected by chemiluminescence using the appropriate HRP-conjugated anti-rabbit or anti-mouse secondary antibody and the Pierce Super Signal™ West Pico detection system.

### MDM2 and mtp53 co-immunoprecipitation from MDA-MB-468 and MDA-MB-468 CRISPR clone G6 (R273Hfs347Δ360-393)

Mouse IgG (Santa Cruz Biotechnology; cat# sc-2025)-protein A/G agarose, 4B2-protein A/G agarose, and SMP14-protein A/G agarose were prepared in siliconized 1.5 ml microfuge tubes by incubating 60 μg antibody with 240 μl of Protein A/G agarose beads (Fisher Scientific; cat# IP1010ML) in a final volume of 1.2 ml of 1 M NaCl, 0.1 M Na-borate pH 8.5 for ≥3 h at 4°C. After binding, antibody/beads were allowed to settle by gravity then centrifuged 1 min 1500 rpm at 4°C. After removal of the supernatant, the antibody beads were successively washed (10 min/wash) with 2× with 1 M NaCl, 0.1 M Na-borate pH 8.5, 2× with PBS, 1× with 1 mg/ml BSA in PBS, and 2× PBS. Following the last wash, the antibody beads were resuspended in 720 μl of PBS (25% slurry); 20 μl of antibody beads (5 μg antibody) was used for each IP reaction. Using the procedure for extract preparation above, one sub-confluent 15 cm plate (∼1 × 10^7^ cells) of each cell line was harvested, washed 2× (20 ml/wash) with ice-cold PBS, and the cell pellets were resuspended in 1.6 ml of IP Buffer (25 mM Tris pH 7.5, 1 mM DTT, 1 mM EDTA, 50 mM NaF, 50 μM NaV, and 10 mM β-glycerolphosphate, 7 mM CHAPS, 10% glycerol, 200 mM NaCl, and complete protease inhibitor cocktail). Cell lysates were incubated on ice for 30 min, clarified by centrifugation at 10 000 rpm at 4°C for 30 min in an Eppendorf 5415R microfuge, and assayed for protein content using the Bio-Rad Bradford Assay. For each extract 1–1.5 mg of total protein was used in each IP reaction (set up in siliconized 1.5 ml of microfuge tubes), which were incubated typically overnight (≤12 h) at 4°C (cold room) with rotation (Labquake; Barnstead Thermolyne). After the incubation, antibody-beads were allowed to settle by gravity, the supernatant removed, 1.4 ml of IP Buffer was added and IP samples washed with rotation in the cold room for 15 min. This wash step was repeated two more times with IP sample collection by centrifugation (4°C for 2 min at 2000 rpm) instead of gravity, and after removal of the last wash, IP samples were resuspended in 54 μl of 1× NuPAGE containing 25 mM DTT, heated at 75°C for 10 min, and then incubated at r.t. for 10 min after addition of iodoacetamide (6 μl). IP samples (12.5% or 25% of each) were subjected to western blot analysis.

### 53BP1 knockdown in T47D

siRNA-mediated 53BP1-depletion was performed using ON-TARGETplus SmartPool TP53BP1 siRNA (Dharmacon; cat# L-003548-00-0005) delivered using the Invitrogen Neon Transfection System as specified by the manufacturer. A total of 1 × 10^6^ cells were transfected with 20 nM siRNA (either control or 53BP1 specific) using the Nucleofector (one 25 ms pulse at 1700 V) and then incubated for 72 h before processing for western blot and PLA analysis.

### Chromatin fractionation assay

Chromatin localization of proteins was measured using the chromatin fractionation assay. Briefly, cell populations (one 50%–60% confluent 10 cm plate/sample) were harvested using a rubber policeman, pelleted and washed with ice-cold PBS, and then resuspended in chromatin fractionation (CF) buffer A (CFA): (10 mM HEPES, 10 mM KCl, 1.5 mM MgCl_2_, 300 mM sucrose, 1 mM DTT, 10% glycerol, 0.1 mM Phenylmethylsulfonyl fluoride (PMSF), 1 μg/ml leupeptin, 1 μg/ml pepstatin A, and 2 μg/ml aprotinin) in a volume of 300 μl (cell density: ∼1 × 10^7^ cells/ml). After hypotonic swelling, cells were then lysed by addition of Triton X-100 (final concentration 0.15%) on ice for 15 min, and the nuclei pelleted by low-speed centrifugation (4 min at 1300 × *g* at 4°C; the supernatant (S1) was saved as cytosolic extract). After washing twice with 300 μl of CFA + 0.15% Triton X-100, nuclei were lysed by resuspending in 300 μl of chromatin fractionation buffer B (CFB): (3 mM EDTA, 0.2 mM ethylene glycol tetraacetic acid (EGTA), 1 mM DTT, 0.1 mM PMSF, 1 μg/ml leupeptin, 1 μg/ml pepstatin A, and 2 μg/ml aprotinin) and incubating on ice for 30 min. Chromatin from each sample was pelleted by centrifugation (1700 *× g* at 4°C for 4 min), washed with 500 μl of CFB twice, and then resuspended in 300 μl of CFB and sheared by sonication. The protein concentration of both S1 and chromatin fractions were quantified using the Bio-Rad Bradford assay and subjected to SDS–PAGE and western blot analysis (7.5 μg of the S1 fraction; 10 μg of the chromatin fraction).

### Chromatin extract-soluble immunoprecipitation of 53BP1 lysates prepared using benzonase

The chromatin fractionation assay (CF) above was used to prepared the chromatin lysates with the following changes: Two 15-cm plates of each cell line (T47D mlp, total ∼3 × 10^7^ cells) were harvested, washed with PBS, and then resuspended in 900 μl of CFA. After hypotonic swelling for 15 min, cells were then lysed by addition of Triton X-100 (final concentration 0.15%) on ice for 15 min, and the nuclei pelleted by low-speed centrifugation (4 min at 1300 × *g* at 4°C; the supernatant (S1) was saved as cytosolic extract). After washing once with 900 μl of CFA supplemented with 10 μM Cathepsin L inhibitor Z-FY-CHO (CPLi) + 0.15% Triton X-100, nuclei were lysed by resuspending in 900 μl of CFB supplemented with 10 μM CPLi on ice for 30 min. The chromatin was pelleted by centrifugation [1700 ×*g* at 4°C for 4 min; supernatant was saved as the nuclear soluble (NS) fraction] and gently resuspended in 400 μl of benzonase digestion buffer A (BBA: 20 mM Tris–HCl pH 8.0, 1 mM DTT, 0.4% Triton X-100, 10% glycerol, 0.1 mM PMSF, 1 μg/ml leupeptin, 1 μg/ml pepstatin A, and 2 μg/ml aprotinin) with 10 μM CPLi, after which MgCl2 was added to a final concentration of 1.5 mM and 125 units of benzonase were added, and the digestion was allowed to proceed for 2.5 h at room temperature with rocking. After incubation, the insoluable material was separated by gravity and the supernatant collected as the soluable fraction and placed on ice. The insoluble material was washed with 1.2 ml of benzonase dilution buffer B (BDB: 20 mM Tris–HCl pH 8.0, 1 mM DTT, 200 mM NaCl, 10% glycerol, 0.1 mM PMSF, 1 μg/ml leupeptin, 1 μg/ml pepstatin A, and 2 μg/ml aprotinin) with 10 μM CPLi for 15 min with rocking at 4°C and then collected by centrifugation. The resulting supernatant was added to the post-benzonase soluble fraction to form the chromatin extract soluble (CES) from which 53BP1 was immunoprecipitated for 4 h using 2.5 μg antibody (53BP1 PLA0303)/10 μl of Protein G Mag Sepharose beads (Cytiva cat# 28951379) and 400 μl of CES at 4°C with rocking. Post incubation, the beads were collected, washed once with 40× the bead volume with IP wash buffer (IPW: 20 mM Tris–HCl pH 8.0, 1 mM DTT, 150 mM NaCl, 0.1% Triton X-100, 10% glycerol, 0.1 mM PMSF, 1 μg/ml leupeptin, 1 μg/ml pepstatin A, and 2 μg/ml aprotinin, 10 μM CPLi) for 15 min at 4°C with rocking, and then bound proteins were eluted by with 40 μl of NuPAGE and processed for electrophoresis and western blot analysis as previously described.

### Image acquisition, processing, and analysis

Sample images were collected using a Nikon A1 confocal microscope (×60/1.45/oil objective), and maximum projection images were created and processed using Nikon Elements NIS software. Objects within images (nuclei, EdU-labeled nuclei, and PLA foci) were identified and quantified using Cell Profiler. Graphical representations of data and statistical analysis (Kruskal–Wallis unpaired one-way ANOVA with Dunn’s correction for three or more samples; Mann–Whitney *U* for two samples) were generated with GraphPad Prism. Mean PLA foci/nucleus (total or EdU-labeled) plus 95% confidence interval are indicated and statistical significance of adjusted *P*-values are denoted as follows: *P* < .05 (*); *P* < .01 (**); *P* < .001 (***); *P* < .0001 (****); *P* > .05 (ns). Representative fields of images prepared using ImageJ. Means are reported in the manuscript, population size of samples are reported within the figure legends, and statistical analysis including SD and SEM for all populations are included at the Zenoda link (see data availability statement).

## Results

### Loss of MDM2 influences the chromatin binding activity of 53BP1

**Figure 1.**
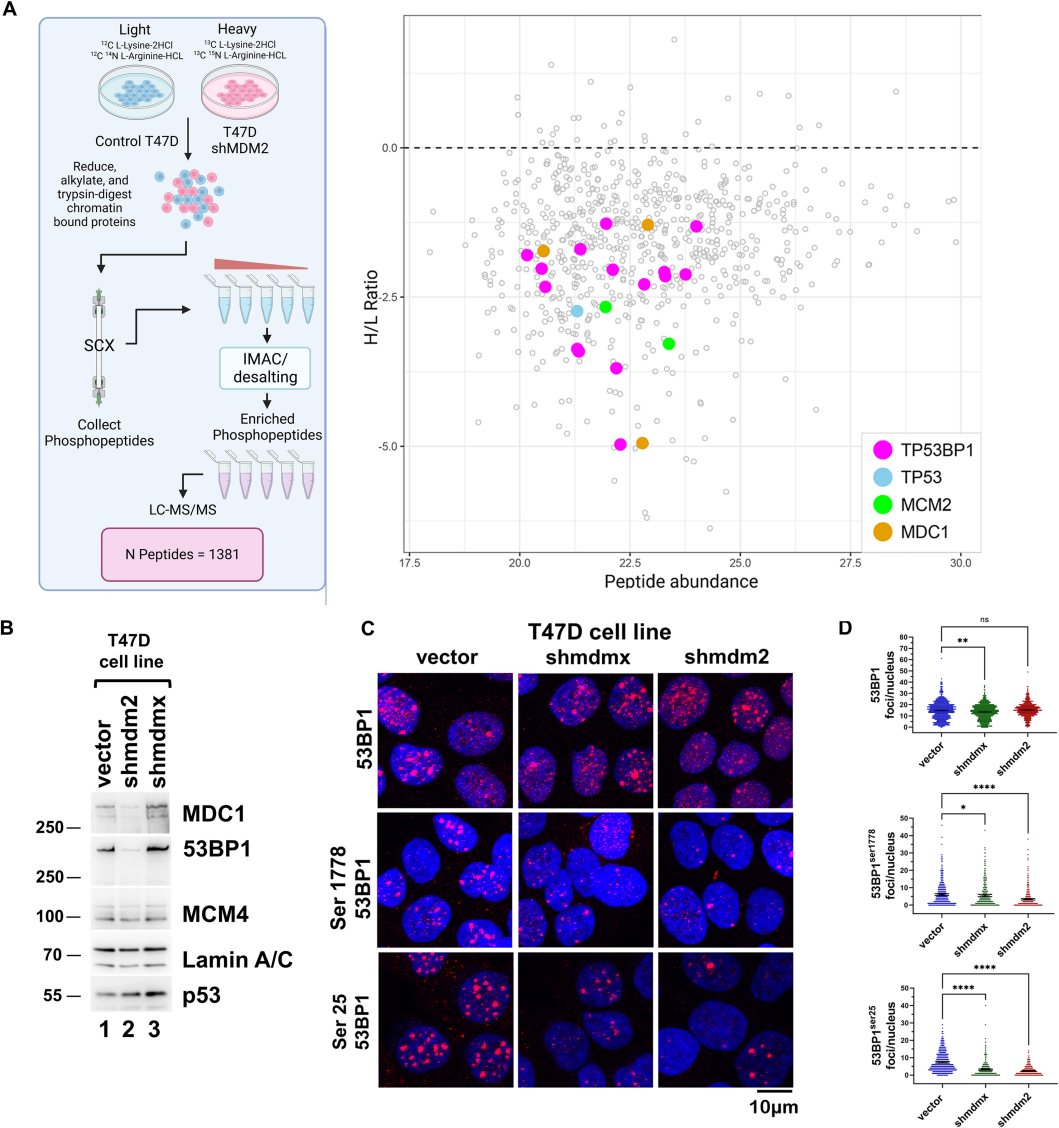
Reduced MDM2 protein in T47D cells causes reduced chromatin phosphoproteins 53BP1 and MDC1. (**A**) Experimental design workflow of the SILAC analysis (created in BioRender. Harmon, K. (2025) https://BioRender.com/oyd7ala). Chromatin isolated from a mixture of T47D vector control cells (MDM2-competent) cultured in natural amino acid medium, and T47Dshmdm2 cells (MDM2-depleted) cultured in heavy isotope amino acid medium, was subjected to proteolysis followed by phospho-peptide purification and enrichment. Scatter plot represents the *H/L* ratio versus abundance of peptides identified by mass spectrometry, with those corresponding to TP53BP1 (magenta), TP53 (blue), MCM2 (green), and MDC1(brown) highlighted. (**B**) Chromatin (5 μg) isolated from T47D vector control, T47Dshmdm2, and T47Dshmdmx cells was subjected to SDS–PAGE/western blot analysis for 53BP1, MDC1, MCM4, lamin A/C, and mtp53. (**C**and**D**) IF of total 53BP1 (i), phospho-53BP1^ser25^ (ii), or phospho- 53BP1^ser1778^ (iii) within T47D vector control nuclei [(i) 528, (ii) 549, and (iii) 501], T47Dshmdm2 nuclei [(i) 549,(ii) 520, and (iii) 501], and T47Dshmdmx nuclei [(i) 623, (ii) 376, and (iii) 501]. Confocal images for six fields for each were acquired and the number of 53BP1 foci per nucleus from each cell population indicated above was determined. Representative data (*n* = 3) with mean, 95% CI, and Kruskal–Wallis statistical significance test prepared as described in the “Materials and methods” section. **** Indicates a p-value less than or equal to 0.0001, ** indicates a p-value less than or equal to 0.01, * indicates a p-value less than or equal to 0.05, and ns is nonsignificant.

Our previous work indicated that MDM2 and MDMX have tumor driving properties even in the presence of mtp53 [37, 41–43]. Therefore, we asked what cellular phospho-proteins were involved in the cancer cell survival pathways. We previously showed that loss of MDM2 impairs T47D and MCF-7 cell proliferation [37, 41–43]. Additionally, we demonstrated that knockdown of MDM2 inhibits triple negative breast cancer MDA-MB-231 and T47D cell metastasis in a xenograft model [37, 41]. To determine the influence of MDM2 on signal transduction at the chromatin protein level we performed stable isotope labeling with amino acids in cell culture (SILAC) in T47D vector control and T47D sh*mdm2* (MDM2-depleted) cells. We purified chromatin-associated phospho-peptides, which were then subjected to LC-MS/MS mass spectrometry analysis (see Fig. 1A for workflow). The analysis revealed 1381 peptides corresponding to 317 unique proteins under-represented on chromatin in MDM2-depleted cells and 20 proteins over-represented (Fig. 1A and https://borreliabase.org/∼wgqiu/clickme-khalikuz/temp-Points.html; the data are available via ProteomeXchange PXD061454). Prominent among the 1381 peptides identified were multiple peptides of 53BP1 and MDC1, two chromatin associated factors mobilized early in the DDR to genotoxic stress. They function together to demarcate double-strand breaks (DSBs) in chromatin domains marked by post-translationally modified forms of H2AX [44–46]. Given that 53BP1 participates in a signaling axis with p53 [12], and MDM2 interacts with MDC1 (also called NFBD1) [47], we asked if 53BP1 chromatin localization required the MDM2 protein.

We examined the validity of the SILAC data by two methods using T47D vector control (T47D vector), *shmdm2* (MDM2-depleted), and *shmdmx* (MDMX-depleted) cells. We compared the 53BP1 and MDC1 protein levels tightly bound to isolated chromatin by western blot analysis (Fig. 1B), and immunofluorescence for total 53BP1, phospho-Ser 25 53BP1, and phospho-Ser1778 53BP1 (Fig. 1C and D). Consistent with the SILAC data, we found reduced levels of 53BP1 and MDC1 associated with MDM2-depleted cell chromatin compared to the vector (Fig. 1B, compare lanes 1 and 2), and reduced levels of phospho-Ser 25 53BP1, and phospho-Ser1778 53BP1 foci within both MDM2-depleted and MDMX-depleted cells (Fig. 1C and D) compared to the T47D vector control population using immunofluorescence. Interestingly, immunofluorescence did not show significant changes in total 53BP1 levels (as it was a combination of nuclear soluble and chromatin bound populations). However, the influence of MDM2 depletion on phosphorylated 53BP1 was consistent with the chromatin binding activity data as phosphorylation of 53BP1 happens when 53BP1 is bound to chromatin. While there is some reduction of total 53BP1 noted following MDMX depletion, the reduction in phosphorylated 53BP1 was more profound. Thus, together with our SILAC data, the chromatin fractionation and immunofluorescence analyses pointed to a role for MDM2 in regulating 53BP1 recruitment to chromatin in cancer cells.

### MDM2 interacts with 53BP1 *in vivo*

**Figure 2.**
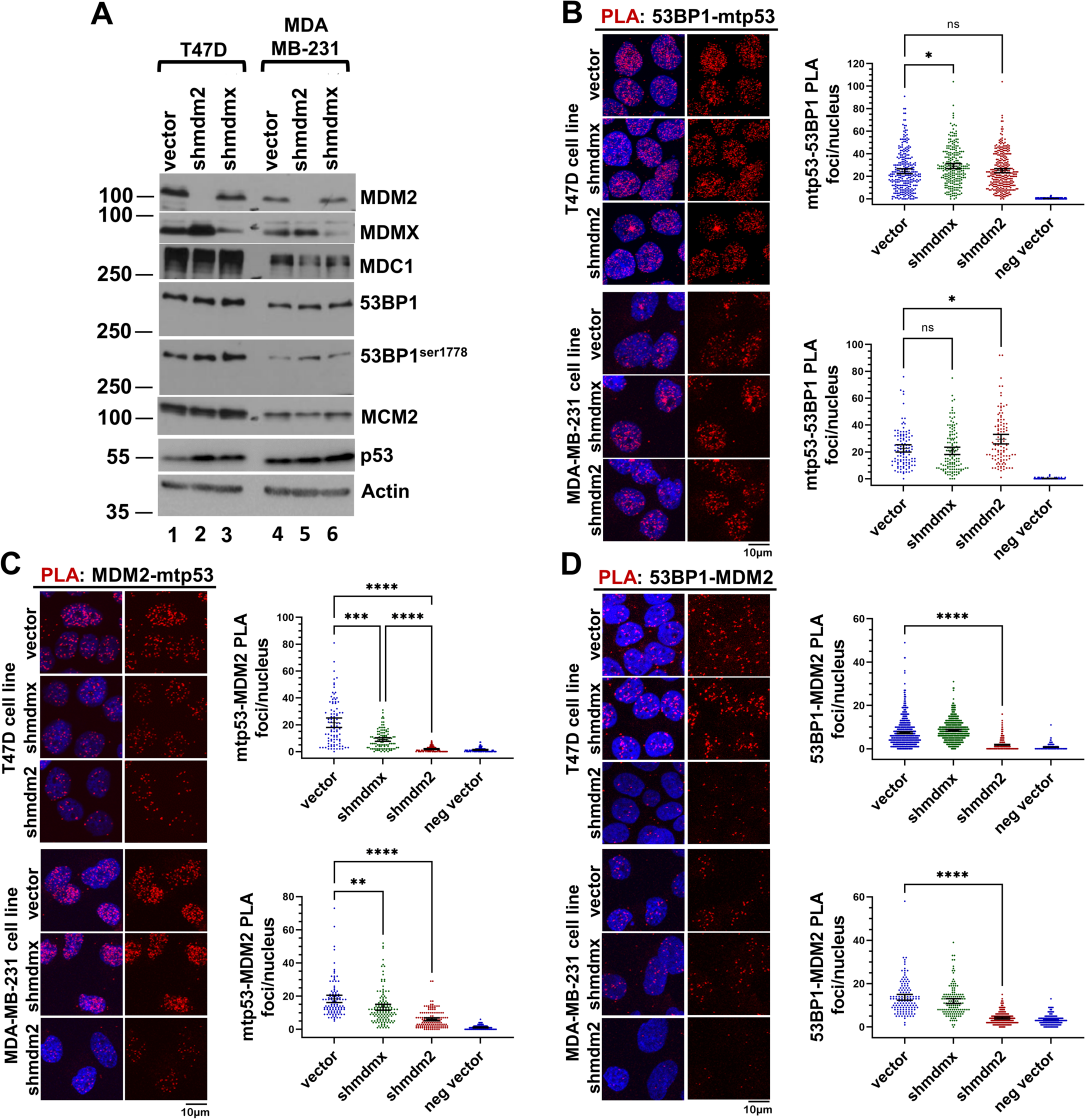
53BP1 in breast cancer cells interacts with both mtp53 and MDM2. (**A**) Relative abundance of mtp53, MDM2, MDMX, 53BP1, and MDC1 within whole cell extracts (20 μg) prepared from T47D (L194F) and MDA-MB-231 (R280K) cell lines determined by SDS–PAGE/western blot analysis. (**B–D**) Association of mtp53, MDM2, and 53BP1 *in vivo* measured using the PLA. PLA analyses of mtp53-53BP1 (panel B), MDM2-mtp53 (panel C), and MDM2-53BP1 (panel D) within T47D and MDA-MB-231 cells were performed as described in the “Materials and methods” section; primary antibodies are PLA rabbit anti-p53, PLA goat anti-53BP1, and mouse anti-MDM2 4B2. Confocal images for 4–6 fields for each were acquired and the number of PLA foci per nucleus for each cell population was determined (*n* = 3 for T47D, *n* = 2 for MDA-MB-231). Representative data with mean, 95% CI, and Kruskal–Wallis statistical significance test prepared as described in the “Materials and methods” section from the indicated number of cells: Panel B vector (T47D = 276; MDA-MB-231 = 108), shmdmx (T47D = 239; MDA-MB-231 = 128), and shmdm2 (T47D = 310; MDA-MB-231 = 106); Panel C vector (T47D = 92; MDA-MB-231 = 104), shmdmx (T47D = 108; MDA-MB-231 = 131), and shmdm2 (T47D = 101; MDA-MB-231 = 136); Panel D vector (T47D = 761; MDA-MB-231 = 129), shmdmx (T47D = 529; MDA-MB-231 = 142), and shmdm2 (T47D = 964; MDA-MB-231 = 163). **** Indicates a p-value less than or equal to 0.0001, ** indicates a p-value less than or equal to 0.01, * indicates a p-value less than or equal to 0.05, and ns is nonsignificant.

We tested the interaction between MDM2 and 53BP1 *in vivo* in two different breast cancer cell lines, T47D and MDA-MB-231 (both which express mtp53 protein). We used the PLA in vector control, MDM2-depleted, and MDMX- depleted derivatives to ensure foci observed were MDM2-dependent. The T47D cells express higher MDM2 levels than MDA-MB-231 cells due to the presence of the *MDM2* gene *SNP309 G/G* allele [48]. Western blot analysis for comparison of protein expression levels in the six cell lines confirmed the higher MDM2 expression in T47D vector control lines and confirmed the depletion of MDM2 and MDMX proteins in the isogenic clones (Fig. 2A). Because p53 is known to interact with MDM2 and 53BP1, we tested the ability of mtp53 to form PLA foci with either 53BP1 or MDM2. In both T47D and MDA-MB-231 vector control populations, we observed PLA foci (red) indicative of 53BP1–mtp53 interactions (Fig. 2B) and MDM2–mtp53 interactions (Fig. 2C). In contrast to the 53BP1–mtp53 PLA interactions that were similar in all three variants of each cell line (Fig. 2B), the MDM2–mtp53 PLA interactions were less abundant in the MDMX-depleted populations (Fig. 2C). The mean MDM2–mtp53 PLA foci/nucleus in vector control T47D was 21.5 and in MDMX-depleted T47D reduced to 9.0; the mean MDM2–mtp53 PLA foci/nucleus in vector control MDA-MB-231 was18.35 and in MDMX-depleted T47D reduced to 13.3. Thus, the 53BP1–mtp53 interaction appeared MDM2- and MDMX-independent, whereas the MDM2–mtp53 interaction was enhanced by MDMX, an observation consistent with previous studies of the p53-MDM2/MDMX complex. Importantly, we detected a significant 53BP1–MDM2 PLA interaction in both cell lines (Fig. 2D) that was neither diminished nor enhanced in the MDMX-depleted population. This suggested both forms of MDM2 (homodimer and heterodimer) are competent in this assay. We confirmed the specificity of the 53BP1 PLA antibody used in all our experiments by conducting PLA analyses for 53BP1–MDC1, 53BP1–mtp53, and 53BP1–MDM2 within T47D vector and T47D.*shmdm2* cell lines in which 53BP1 was depleted using siRNA to 53BP1 (Supplementary Fig. S1, see panel A for western blot and B–D for the 53BP1–MDC1, 53BP1–mtp53, and 53BP1–MDM2 PLA results).

### The C-terminus of mtp53 is required for MDM2 interaction with 53BP1

**Figure 3.**
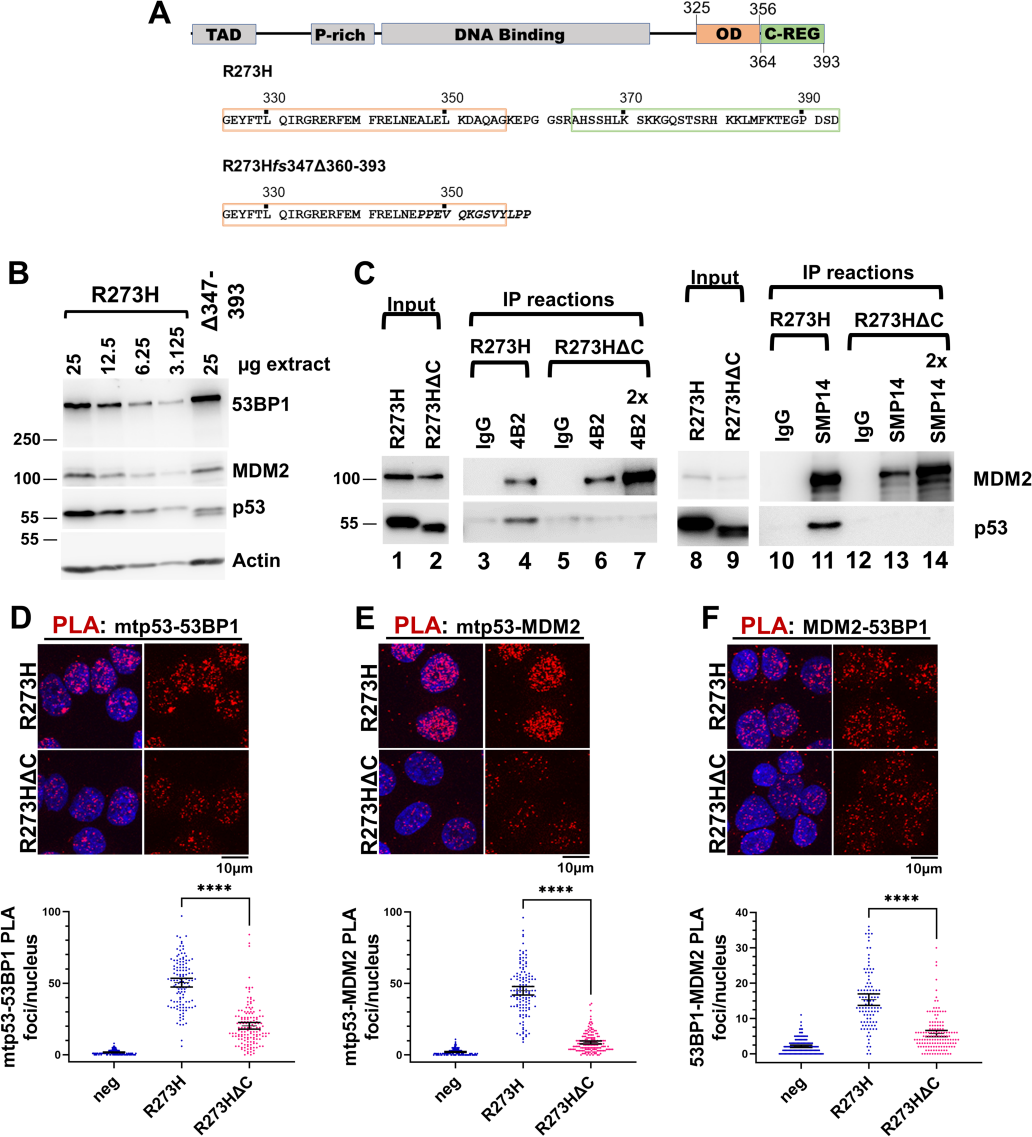
The MDM2–53BP1 interaction is promoted by the mtp53 C-terminus. (**A**) The C-terminus of mtp53 R273H within MDA-MB-468 was modified using CRISPR–Cas9 to create the cell line MDA-MB-468 R273Hfs347Δ360-393 (termed G6; mtp53 derivative R273HΔC). (**B**) Relative protein levels of 53BP1, MDM2, and mtp53 within MDA-MB-468 (25, 12.5, 6.25, and 3.125 μg) and G6 (25 μg) cell lines was examined by SDS–PAGE/western blot analysis. (**C**) Loss of mtp53 C-terminus disrupts mtp53 co-IP with MDM2. Extracts from MDA-MB-468 and G6 cell lines were incubated with either normal mouse IgG (negative control) or anti-MDM2 antibodies 4B2 (lanes 1–7) or SMP14 (lanes 8–14), and IP reactions were examined for MDM2 and mtp53 by western blot analysis. Lanes 1–7: 4B2 IP reactions from 800 μg of extract (input); lanes contain 10% of total IP and 2% of input. Lanes 8–14: SMP14 IP reactions from 1600 μg of extract; lanes contain 12.5% each IP and 0.5% of input. Lanes labeled 2× (lane 7 for the 4B2 IPs and lane 14 for the SMP14 IPs) contain twice the amount of the G6 extract MDM2 IP; a lighter exposure of mtp53 input is presented due to the vast excess of mtp53 compared to MDM2 within the MDA-MB-468 cell lines. (**D–F**) PLA analysis of mtp53–53BP1 (panel D), MDM2–mtp53 (panel E), and MDM2–53BP1 (panel F) in MDA-MB-468 and G6. PLA analysis of the indicated proteins was measured using PLA rabbit anti-p53, PLA goat anti-53BP1, and mouse anti-MDM2 2A9 antibodies. Confocal images for 3–5 fields for each were acquired and the number of PLA foci per nucleus for each cell population was determined (*n* = 2). Representative data with mean, 95% CI, and Kruskal–Wallis statistical significance test prepared as described in the “Materials and methods” section from the indicated number of cells in panel: (D) R273H = 120; R273HΔC = 144; (E) R273H = 131; R273HΔC = 159; (F) R273H = 102; R273HΔC = 138. **** Indicates a p-value less than or equal to 0.0001.

To address the possibility that mtp53 coordinated the MDM2–53BP1 PLA interaction, we carried out PLA analysis of MDM2-53BP1 within the MDA-MB-468 (mtp53 R273H) breast cancer cell line and an MDA-MB-468 derivative in which CRISPR-Cas9 was used to delete the mtp53 C-terminal domain that interacts with MDM2 (Fig. 3A) [36, 39]. The resulting variant mtp53 R273H*fs*347Δ360–393 protein (referred to here as R273HΔC) lacks the C-terminal 10 amino acids of the oligomerization domain and the entire C-terminal regulatory domain, which includes a functional MDM2-binding site [49]. We reasoned that a weakened R273H-MDM2 complex caused by loss of the C-terminus would correlate with a reduction in the MDM2-53BP1 PLA foci in the variant R273HΔC-expressing cells. Western blot analysis of extracts from MDA-MB-468 parental and the CRISPR–Cas9 derivative showed comparable levels of 53BP1 and MDM2, ∼3-fold lower levels of the R273HΔC variant protein compared to unaltered R273H mtp53 (Fig. 3B). We asked if R273HΔC was in a complex with MDM2 by co-IP analysis with two different MDM2 antibodies, 4B2 and SMP14 (Fig. 3C). We found that although both MDM2 antibodies pulled down unaltered R273H from the parental cell line extract (Fig. 3C, 4B2 IP lane 4; SMP14 IP lane 11), we were unable to detect the variant R273HΔC within either the 4B2 IP or SMP14 IP from the CRISPR derivative cell line extract (Fig. 3C, 4B2 IP lanes 6 and 7; SMP14 IP lanes 13 and 14). This indicated that loss of the C-terminus of mtp53 R273H prevented stable binding of mtp53 to MDM2.

We compared the PLA analyses for mtp53–53BP1, mtp53–MDM2, and MDM2–53BP1 in the MDA-MB-468 cell lines expressing either unaltered R273H- and the variant R273HΔC (Fig. 3. panels D–F). Whereas a 2.5-fold reduction in the mean mtp53-53BP1 PLA foci/nucleus was observed in the C-terminal deletion population compared to the parental R273H (Fig. 3D, mtp53–53BP1 PLA, R273H = 50.4; R273HΔC = 20.3), we detected a 5.1-fold reduction in the mean mtp53–MDM2 PLA foci/nucleus in the C-terminal deletion R273HΔC population compared to the parental cells (Fig. 3E, mtp53–MDM2 PLA R273H = 44.9; R273HΔC = 8.8). When taking the semi-quantitative western blot analysis and co-IP experiments presented into account, it was clear the reduced mtp53–MDM2 PLA interaction was the result of a direct protein–protein interaction. A comparison of the MDM2–53BP1 PLA interaction within the two cell lines revealed a 2.7-fold decrease in the mean MDM2–53BP1 PLA foci/nucleus when the mtp53–MDM2 interaction was disrupted (Fig. 3F, MDM2–53BP1 PLA R273H = 15.3 versus R273HΔC = 5.7). Taken together, these data suggest that mtp53 coordinates MDM2 to interact with 53BP1. We dissected this interaction further by using the small molecule p53–MDM2 disrupter Nutlin 3a (N3a), which is known to allow release and activation of wtp53 [49, 50].

### Both the MDM2–mtp53 and MDM2–53BP1 PLA interactions are Nutlin 3a sensitive

**Figure 4.**
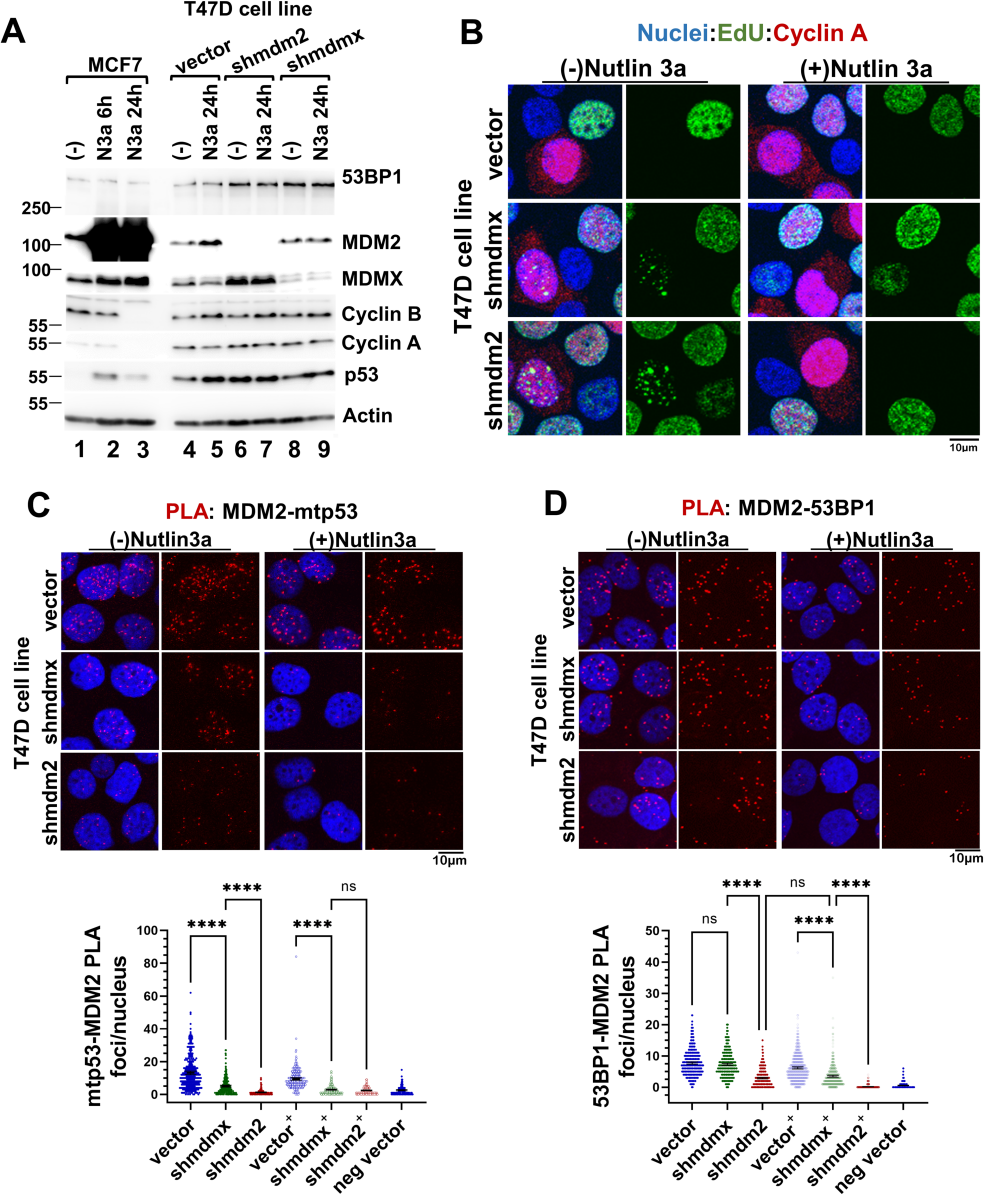
The MDM2-mtp53 and MDM2–53BP1 interactions are Nutlin 3a sensitive. (**A**) Western blot analysis of whole cell extracts (10 μg) from MCF7 and T47D cell line populations treated for the indicated time with either vehicle or 10 μM Nutlin 3a (labeled N3a or “+” in graphs) for the indicated proteins. (**B**) Nutlin 3a does not inhibit T47D cell cycle progression. Twenty-four hour post-treatment with either vehicle or 10 μM Nutlin 3a, cells were labeled with EdU for 20 min and assayed for Cyclin A2 by immunofluorescence. Confocal images from at least three fields were acquired and the number of EdU + and Cyclin A + nuclei were quantified (tabulated in S4, panel C) in each population of vehicle-treated [vector = 289; shmdmx = 278; shmdm2 = 249] and Nutlin 3a-treated [vector = 227; shmdmx = 283; shmdm2 = 245] cells. The S/G2 fraction (total Cyclin A + nuclei) for vehicle-treated: vector = 33.6%, shmdmx = 37.8%, shmdm2 = 36.8%; Nutlin 3a-treated: vector = 38.3%, shmdmx = 27.0%, shmdm2 = 29.4%. (**C**and**D**) Nutlin 3a disrupts mtp53–MDM2 and 53BP1–MDM2 PLA foci in T47D. Twenty-four hour post-treatment with vehicle or 10 μM Nutlin 3a cell populations PLA interactions were measured between MDM2–mtp53 (panel C) and MDM2–53BP1 (panel D) using PLA rabbit anti-p53, PLA goat anti-53BP1, and mouse anti-MDM2 4B2 primary antibodies. Confocal images for 4–6 fields for each were acquired and the number of PLA foci per nucleus from each cell population was determined (*n* = 2 for panel C and *n* = 3 for panel D). Representative data with mean, 95% CI, and Kruskal–Wallis statistical significance test prepared as described in the materials and methods from the indicated number of cells in panel: (C) vehicle-treated [vector = 328; shmdmx = 298; shmdm2 = 249], Nutlin 3a-treated [vector = 323; shmdmx = 364; shmdm2 = 285] and (D) vehicle-treated [vector = 543; shmdmx = 548; shmdm2 = 455], Nutlin 3a-treated [vector = 610; shmdmx = 545; shmdm2 = 618]. **** Indicates a p-value less than or equal to 0.0001.

The MDM2–53BP1 PLA analyses within C-terminal deletion mtp53 R273H (MDM2-binding deficient) and parental R273H (MDM2-binding proficient) MDA-MB-468 suggested that mtp53 directed MDM2 into proximity with 53BP1. We therefore asked if we could phenocopy the reduced MDM2–53BP1 PLA observation by treating T47D cells with the p53–MDM2 interaction disruptor N3a (Fig. 4). Western blot analysis of extracts from N3a-treated MCF7 cells demonstrated that N3a activated the wtp53 pathway (Fig. 4A, lanes 1–3). Comparison of MCF7 and T47D populations revealed opposite portraits of MDM2, and the S-G2/M cyclins Cyclin A and B (Fig. 4 A, compare lanes 1–3 to lanes 4–9). In contrast, extracts from untreated and N3a-treated T47D cell populations contained similar levels of MDM2 and both Cyclin A and B proteins (Fig. 4A, lanes 4–9). We next asked by immunofluorescence if the observed Cyclin A protein levels correlated with the %S-G2 cells in cell populations 24 h post Nutlin 3a exposure. Untreated and N3a-treated T47D populations examined for EdU incorporation and Cyclin A2 expression, and confocal images were scored for the number of EdU+/Cyclin A + and EdU-/Cyclin A + nuclei (Fig. 4B for imaging and tabulation of EdU+/Cyclin A + and EdU-/Cyclin A + T47D nuclei presented in Supplementary Fig. S2A). Consistent with the western blot analysis, the %S-G2 for the T47D untreated and N3a-treated cell populations was not significantly different. We therefore concluded that Nutlin 3a treatment of mtp53- expressing T47D cells did not induce a measurable cell cycle arrest phenotype.

We reasoned that T47D cells post 24 h treatment with Nutlin 3a would demonstrate MDM2–mtp53 (Fig. 4C) and MDM2–53BP1 PLA reduction (Fig. 4D). Using the PLA assay, we recapitulated the published *in vivo* effect of N3a, observing significant reduction in the mtp53–MDM2 interaction (Fig. 4C, see N3a treatment denoted by +). The vector control mean mtp53–MDM2 PLA foci/nucleus reduced from 13.1 to 9.4, and in the MDMX-depleted population the mean mtp53–MDM2 PLA foci/nucleus reduced from 5.1 to 2.9. The MDMX-depleted population result provided further confirmation of the contribution of MDMX to the stability of the MDM2–p53 complex (Fig. 4C). Examination of the MDM2–53BP1 PLA interaction within N3a-treated populations yielded similar reduction results, with the mean MDM2–53BP1 PLA foci/nucleus in the treated compared to untreated T47D vector population being significantly lower (Fig. 4D). This effect was striking in the MDMX-depleted T47D population, with the mean MDM2–53BP1 PLA foci/nucleus in the untreated reducing from 7.3 and to 3.5. The reduction was to a level comparable to that of the untreated MDM2-depleted population which was 2.9 and even this was able to be reduce by N3a treatment to a value of 0.2 (Fig. 4D). Moreover, the reduction in MDM2–53BP1 foci could not be attributed to inhibition of the mtp53–53BP1 PLA interaction, as we found comparable mtp53–53BP1 PLA foci/nucleus within vector and MDM2-depleted untreated and N3a-treated populations (Supplementary Fig. S2B). Taken together, this suggests that a chromatin-associated mtp53–MDM2 complex forms (stabilized by MDMX) and this in turn regulates 53BP1 on the chromatin.

### MDM2 regulates the interaction between 53BP1 and MDC1

**Figure 5.**
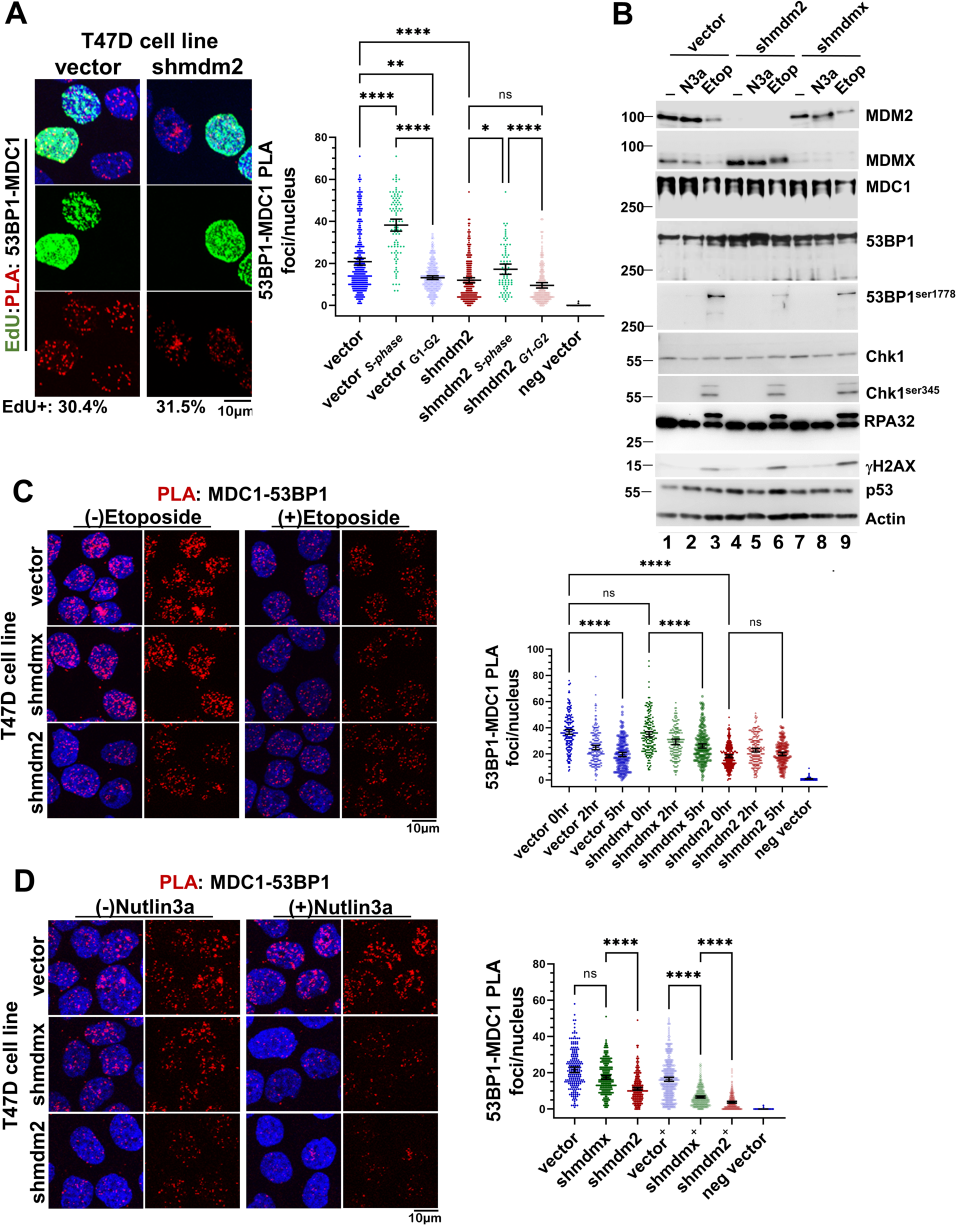
MDM2 promotes 53BP1–MDC1 complex formation. (**A**) Reduced 53BP1–MDC1 PLA foci in T47D lacking MDM2. EdU-labeled T47D vector and shmdm2 cells were assayed for 53BP1–MDC1 PLA foci using PLA goat anti-53BP1 and PLA rabbit anti-MDC1 antibodies. Shown under representative images (*n* = 3) is the number of EdU + nuclei identified within each cell population and graphed is the number of PLA foci per nucleus. For the PLA analysis representative data with mean, 95% CI, and Kruskal–Wallis statistical significance test prepared as described in the “Materials and methods” section from the indicated number of cells: vector (total = 329, S-phase = 100, G1-G2 = 229); shmdm2 (total = 251, S-phase = 79, G1-G2 = 172). (**B**) Activation of the DDR in T47D cell lines by Etoposide but not Nutlin-3a. Whole cell extracts (20 μg) from T47D cell lines treated with either 50 μM Etoposide for 5 h (Etop), or 10 μM Nutlin 3a for 24 h (N3a) were analyzed for the indicated proteins by WB. (**C** and **D**) MDC1–53BP1 PLA foci are disrupted by both DDR activation and Nutlin 3a. MDC1–53BP1 PLA analyses were performed within each T47D cell line at the indicated time points post Etoposide treatment (panel C) or 24 h-post Nutlin 3a treatment (panel D) using PLA goat anti-53BP1 and PLA rabbit anti-MDC1. Confocal images for 3–6 fields for each were acquired and the number of PLA per nucleus from each cell population was determined (*n* = 3 for panel C and *n* = 3 for panel D). Representative data with mean, 95% CI, and Kruskal–Wallis statistical significance test prepared as described in the “Materials and methods” section from the indicated number of cells in panel: (C) vehicle-treated (0 h Etop) [vector = 208; shmdmx = 198; shmdm2 = 247], 2 h Etoposide [vector = 205; shmdmx = 184; shmdm2 = 231], 5 h Etoposide [vector = 189; shmdmx = 224; shmdm2 = 176]; (D) vehicle-treated [vector = 198; shmdmx = 270; shmdm2 = 336] and Nutlin 3a-treated [vector = 380; shmdmx = 339; shmdm2 = 252]. **** Indicates a p-value less than or equal to 0.0001, ** indicates a p-value less than or equal to 0.01, * indicates a p-value less than or equal to 0.05, and ns is nonsignificant.

There are many proteins that influence 53BP1 DDR activity, and the protein MDC1 is critical [51]. MDC1 aids 53BP1 recruitment to sites of damaged chromatin. Given that our SILAC screen identified MDC1 as a potential MDM2-regulated chromatin associated protein, we tested if 53BP1–MDC1 interactions were MDM2 dependent (Fig. 5). We determined, by 53BP1–MDC PLA in EdU-labeled T47D vector and MDM2-depleted populations, that more 53BP1–MDC1 PLA foci existed in the vector control compared to the MDM2-depleted population (Fig. 5A, with average 53BP1–MDC1 PLA foci/nucleus in vector cells being 20.8; while in T47D.*shmdm2* the average 53BP1–MDC1 PLA foci/nucleus was 12). The highest 53BP1–MDC1 PLA foci/nucleus levels were observed in EdU-positive (labeled S-phase vector cells 53BP1–MDC1 PLA being 36.7 and *mdm2-*depleted cells reducing to 17.2) compared to EdU-negative nuclei (labeled G1-G2, vector cells being 13.2 and *mdm2-*depleted cells being 9.6). In addition, 53BP1–MDC1 PLA analysis of cell populations pre-extracted with cytoskeletal buffer (CSK) before fixation indicated biochemically distinct MDM2-dependent MDC1–53BP1 PLA interactions in the CSK extraction-resistant, stable chromatin-bound MDC1–53BP1 complexes (Supplementary Fig. S3A and B). To determine if MDM2 required either DDR activity or mtp53 for the promotion of MDC1–53BP1 complex formation, we performed MDC1–53BP1 PLA analysis on T47D cells under three different experimental conditions. The first was etoposide induced DNA damage, the second was disruption of the p53-MDM2 binding by N3a or ALRN-6924, and the third was inhibition of ATM activity by KU-55933 (ATMi).

We determined the influence of either 10 μM N3a for 24 h or 50 μM etoposide (Etop) for 5 h, on the presence of phospho-Ser1778 53BP1, γH2AX, phospho-Chk1, and the levels of MDM2, MDMX, and mtp53 (Fig. 5B). Only the etoposide-treated cell extracts demonstrated increased levels of DDR pathway markers (Fig. 5B, compare lanes 3, 6, and 9 with untreated lanes 1, 4, and 7), and a reduction in both MDM2 and MDMX (Fig. 5B, compare lanes 3 and 9). It is important to note that the loss of the MDMX protein was dependent on MDM2 function (Fig. 5B, lane 6). In contrast, no change in the levels of DDR markers was observed within N3a-treated extracts (lanes 2, 3, and 8).

The MDC1–53BP1 PLA analysis at 2 h, and 5 h, post-etoposide treatment revealed a reduction over time in the average number of MDC1-53BP1 foci/nucleus in both the vector control and MDMX-depleted populations (Fig. 5C). We also noted that etoposide treatment of MDM2-depleted cell populations did not reduce their already low number of MDC1–53BP1 PLA foci/nucleus. The mean MDC1–53BP1 PLA foci per nucleus for 0hr time pt in T47D vector was 36.75, in T47D.*shmdmx* was 35, and in T47D.*shmdm2* was to 18.2. The 5hr etoposide treatment mean MDC1–53BP1 PLA foci/nucleus in T47 vector was reduced from 36.75 to 19.5, while in T47D.*shmdmx* cells it was reduced from 35 to 26, and in T47D.*shmdm2* it was increased from 18.2 to 20 (Fig. 5C). This reduction in foci in the vector and MDMX-depleted populations, but not in the MDM2-depleted population, correlated with loss of MDM2 (which clearly was already present in the T47D.*shmdm2* cells). Interestingly, N3a-treated vector control and MDMX-depleted cell populations also displayed a significant reduction in mean MDC1–53BP1 PLA foci per nucleus compared to the untreated populations (Fig. 5D). The mean MDC1–53BP1 PLA foci per nucleus in untreated vector cells was 21.65, while in T47D.*shmdm2* it was 11.1, and in T47D.*shmdmx* it was 17.4 compared to treated MDC1–53BP1 PLA foci per nucleus in vector which was 16.4; in T47D.*shmdm2* it was 3.7, and in T47D.*shmdmx* it was 6.6 (Fig. 5D). However, unlike the response to etoposide, neither DDR activation nor MDM2 downregulation correlated with the observed reduction in MDC1–53BP1 PLA foci in response to N3a treatment (Fig. 5B). Instead, the N3a response mirrored that obtained from the PLA analyses of mtp53–MDM2 and MDM2–53BP1 in T47D, which together indicate disruption of mtp53–MDM2 binding compromises the MDM2–53BP1 interaction. We therefore posit that N3a inhibits mtp53 targeting of MDM2 to 53BP1 and, as a consequence, MDM2-dependent MDC1–53BP1 complex formation.

**Figure 6.**
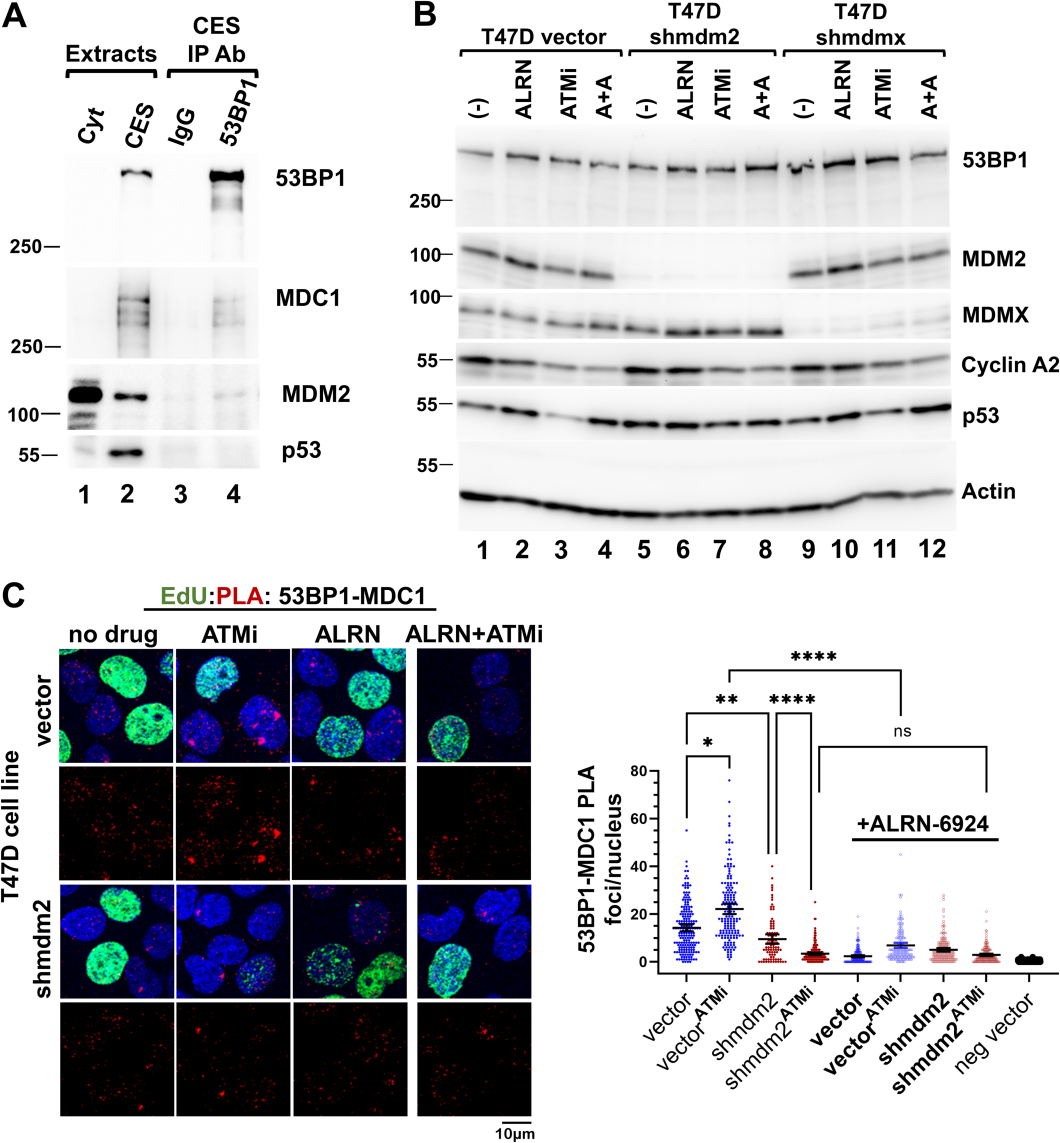
Co-IP demonstrates a 53BP1–MDC1–MDM2 multiprotein complex. (**A**) Co-IP demonstrates a 53BP1–MDC1–MDM2 multiprotein complex. 53BP1 was immunoprecipitated from T47D CES as described in the “Materials and methods” section subjected to western blot analysis for 53BP1, MDC1, MDM2, and p53. Lanes are as follows: (1) cytoplasmic extract, (2) CES IP input, (3) IP with IgG, and (4) IP for 53BP1. (**B**) Inhibition of ATM promotes MDM2 activity. Western blot analysis for the indicated proteins within extracts (10 μg) from T47D populations treated for 24 h with either vehicle,10 μM ALRN-6924 (MDM2/X dual inhibitor), 10 μM KU-55933 (ATMi; ATM inhibitor) or both. (**C**) The MDM2 inhibitor ALRN-6924 reduces whereas the ATM inhibitor increases MDC1–53BP1 PLA foci in T47D. Twenty-four hour post treatment with either vehicle,10 μM ALRN-6924, 10 μM KU-55933 (ATMi) or both T47D vector and shmdm2 cells were labeled with EdU for 20 min and then assayed for 53BP1–MDC1 PLA foci using PLA goat anti-53BP1 and PLA rabbit anti-MDC1 antibodies. Confocal images for several fields were acquired and the number of PLA foci/nucleus from each cell population was determined. Representative data with mean, 95% CI, and Kruskal–Wallis statistical significance test prepared as described in the “Materials and methods” section from the indicated number of cells in: vehicle-treated [vector = 186; shmdm2 = 87], ATMi-treated [vector = 163; shmdm2 = 188], ALRN-6924-treated [vector = 196; shmdm2 = 176], ALRN-6924 + ATMi-treated [vector = 154; shmdm2 = 159]. **** Indicates a p-value less than or equal to 0.0001, ** indicates a p-value less than or equal to 0.01, * indicates a p-value less than or equal to 0.05, and ns is nonsignificant.

We found that etoposide-induced DDR disrupted MDC1–53BP1 PLA foci (Fig. 5A), but the possibility remained that endogenous genotoxic stress and DNA damage promotes MDM2-dependent MDC1–53BP1 complex assembly. Moreover, we observed a trend in increased 53BP1 proteins in the whole cell extracts when either MDM2 or MDMX was depleted (Fig. 5B). The stability of 53BP1 is inhibited by the cysteine protease cathepsin L [52, 53]. The variability in detecting stable 53BP1 was particularly apparent for IP reactions from chromatin fractions unless we added a cathepsin L inhibitor Z-FY-CHO (CPLi) (see CES methods). We designed a protocol for chromatin extraction which enabled stabile 53BP1 for IP. Along with 53BP1 we were able to Co-IP both MDC1 and MDM2 (Fig. 6A). The Co-IP orthogonal approach to the PLA validated the interaction of 53BP1 with both MDC1 and MDM2. PLA detects protein–protein interactions if the two proteins are at least within a 40 nm distance. The PLA method is highly sensitivity but does not characterize how tight the protein–protein interaction is, if it is direct, or if large protein complexes are occurring. Using Co-IP, we demonstrated a reproducible interaction between 53BP1, MDC1, and MDM2 (Fig. 6A). This highlights the fact that the interaction can be observed by PLA and is also tight enough to be detected by Co-IP. Surprisingly we did not detect mtp53 pulled down with the 53BP1 antibody. The mtp53 may be part of weak scaffold for this interaction that is detectable by PLA or it part of an alternative complex not detected by the 53BP antibody. Taken together, the data support a MDM2 scaffold working to facilitate the interaction of 53BP1 with MDC1.

We also tested the ability of MDM2 to act as a scaffold for MDC1–53BP1 PLA foci 24 h after exposure of T47D cells to 10 μM KU-55933 (ATMi), or ATMi plus the dual MDM2/X chemical inhibitor ALRN-6924 (ALRN) (Fig. 6). We assessed the T47D cell extract for protein levels of 53BP1, MDM2, MDMX, Cyclin A2, and mtp53 following drug treatment (Fig. 6B). A reduction in Cyclin A2 levels was observed in extracts from ATMi-treated cell populations suggesting a G1/S block to cell cycle progression (Fig. 6B); untreated and ALRN only extracts lanes 1, 2, 5, 6, 9, and 10; ATMi only and ATMi + ALRN extracts lanes 3, 4, 7, 8, 11, and 12). We also quantified the EdU/Cyclin A positive cells in T47D and MCF7 cell populations treated with either ALRN, ATMi, or ALRN + ATMi (Supplementary Fig. S4). Surprisingly, the ATMi-treated T47D vector control cells showed reduced mtp53 levels (Fig. 6B; compare lane 1 to lane 3). However, the ATMi treatment of T47D.*shmdm2* cells maintained similar mtp53 levels (Fig. 6B, compare lane 5 to lane 7) and treated T47D.*shmdmx* cells showed slight mtp53 reduction (Fig. 6B, compare lane 9 to lane 11). We detected ATMi combined with ALRN abolished the mtp53 reduction (Fig. 6B, compare lane 3 to 4 and lane 9 to 10). As such, we uncovered a latent capacity for MDM2-mediated mtp53 degradation. We examined MDC1–53BP1 PLA foci in ATMi treated cells and observed an increase, that in MDM2-depleted cells was reduced. Furthermore, treatment with ALRN plus or minus ATMi showed similar MDC1–53BP1 reduced foci (Fig. 6C). Thus, ALRN treatment phenocopies N3a inhibition, supporting that MDM2 works with mtp53 to promote 53BP1–MDC1 foci (Figs 6C and 5D). Collectively, these data indicated that a population of MDC1–53BP1 complexes is formed in a mtp53–MDM2-dependent pathway, for which ATM activity is dispensable. As such, we considered the possibility that multiple replication stress pathways crosstalk for mt53 scaffold regulation. Compromised processing of replication intermediates recruits factors that prohibit DNA degradation such as 53BP1 and MDC1, and those that facilitate repair such as the DNA-binding protein PARP1. Both wtp53, and MDM2 (in the context of wtp53) have been shown to coordinate with PARP1 to promote completion of chromosome replication [31]. Furthermore, mtp53 coordinates with PARP1 on replicating DNA [24]. Therefore we asked if the PARP replication stress pathway was also modulated by the mtp53–MDM2 scaffold.

### MDM2 protein reduction increased parylation of chromatin-associated proteins

**Figure 7.**
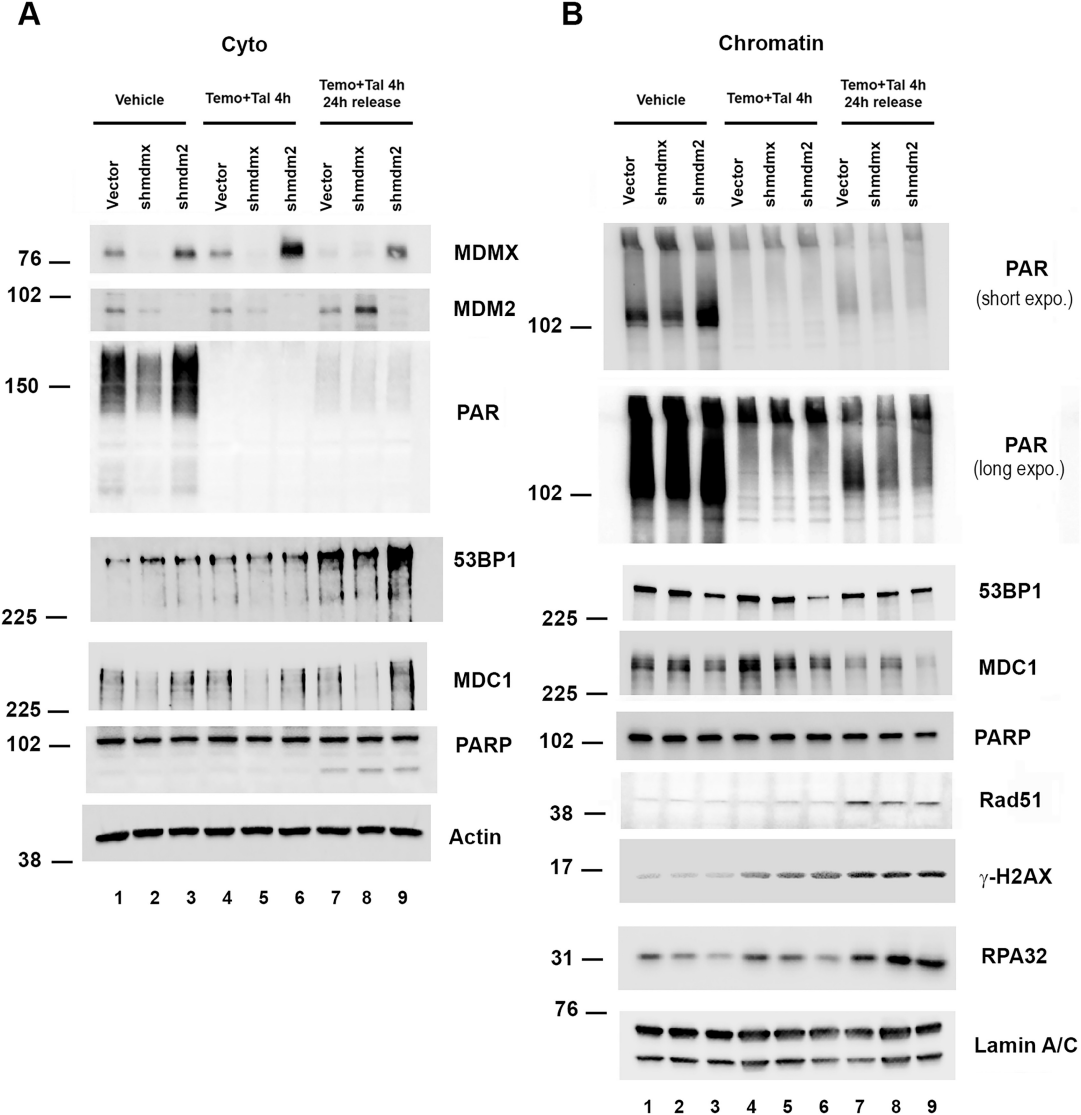
Depletion of MDM2 increases poly(ADP-ribose) modification (PARylation) levels of chromatin bound proteins. Cytosolic (**A**) and chromatin (**B**) fractions were prepared from T47D cells with constitutive shmdm2, shmdmx, or mir30 shRNA-expressing vector cells treated with either vehicle (DMSO), or a combination of 1 mM temozolomide plus10 μM talazoparib (Temo + Tal) for 4 h, or combination of 1 mM temozolomide plus10 μM talazoparib (Temo + Tal) for 4 h and then replaced with fresh media for an additional 24 h. Ten micrograms of cytosolic or chromatin protein was loaded on a SDS–PAGE and protein levels were determined by western blot analysis using the indicated antibodies.

We previously published that mtp53 interacts with PARP1 in breast cancer cells and that mtp53 expression increases cancer cell sensitivity to combination PARP inhibitor (PARPi) talazoparib (Tal) with temozolomide (Temo) treatment [22, 23, 54, 55]. We were curious if the mitigation of replication stress for CPR made use of alternating DNA repair programs when one was disabled. As such, we asked if inhibition of the MDM2–mtp53 interaction influenced either PARP1 levels or PARP activity by investigating total PARylated proteins when MDM2 was depleted. MDM2 targets PARP for ubiquitination and degradation in a wtp53 context [31]. Therefore, we compared MCF7 wtp53 expressing cells to T47D mtp53 expressing cell lines treated with N3a (Supplementary Fig. S4A). In contrast to cell extracts derived from N3a-treated MCF7 (in which we observed lower PARP1 protein levels), extracts prepared from N3a-treated T47D cells showed no change in the levels of the PARP1 protein. This indicated that in the T47D cells PARP was available to ribosylate target proteins. We then proceeded to assess T47D mtp53 expressing cells for the influence of depletion of either MDMX or MDM2 on cytosolic and chromatin PARP activity (Fig 7A and B). The three different T47D cell lines were either left untreated, treated with the combination of a PARP inhibitor Tal and DNA damage agent Temo, or treated with the combination of a Temo plus Tal and then allowed 24 h of DNA repair followed by analyzing the cytosolic and chromatin proteins (Fig. 7A and B). In the cytosol the reduction of MDMX corresponded to a decrease in PARylated proteins (Fig. 7A, lane 2) and 4 h of treatment produced a robust decrease in PARylation of cytoplasmic proteins (Fig. 7A, compare PAR for lanes 1 to 3, to lanes 4 to 9). Importantly in the chromatin fractions before treatment we saw that the depletion of MDM2 corresponded with increased PARylated proteins (Fig. 7B, see lane 3) and this was blocked by Tal treatment (Fig. 7B, lanes 4–6). Interestingly when the cells were given 24 h without drug treatment the increased PARylation was partially restored (Fig. 7B, lanes 7–9), with the MDM2 depleted cells showing the highest migrating forms of PARylated proteins (Fig. 7B, lane 9). This correlated with MDM2 depletion maintaining a reduced level of chromatin-associated MDC1 while all three cell lines showed markers of increased stress by high Rad51, γH2AX, and RPA32 chromatin recruitment (Fig. 7B, compare lanes 7–9). We examined a similar drug treatment protocol in the TNBC cell line MDA–MB-231 and observed a similar result, except in this setting both the depletion of MDM2 and MDMX showed a substantial increase in PARylated proteins (Supplementary Fig. S5). Taken together these results suggest that MDM2 works with mtp53 to assist in coordinating the DDRs initiated in cancer cells during DNA replication stress. We posit that this coordination is responsible for the cancer cell CPR response which allows cancers to survive with accumulated DNA damage that would cause most cells to die.

## Discussion

Herein, we show that MDM2 works together with mtp53 (in a number of different breast cancer cell lines) to coordinate the response of normal replication stress in cancer cells using a 53BP1–MDC1 pathway. Previously MDM2 was shown to interact with MDC1 and the Mre11–Rad50–Nbs1 complex (MRN) DNA repair machinery [9, 47, 56]. Before this work, no known connection had been described between MDM2 and 53BP1. Using a SILAC screen we determined that MDM2 was required for chromatin recruitment of phosphorylated MDC1 and 53BP1 (see Fig. 1). Furthermore, we saw a clear mtp53-dependent interaction of MDM2 with 53BP1 (see Figs 2–4).

When double-strand DNA breaks are generated, the damage is repaired by either the nonhomologous end joining (NHEJ) or the homologous recombination (HR) pathway, with 53BP1 blocking HR and promoting NHEJ [57]. The interaction between 53BP1 and MDC1 is required for recruitment of 53BP1 to DNA breaks [51]. Rad51 directs HR which can only occur during S-G2, when cells have generated sister chromatids [58]. In G1 and G2, the 53BP1 protein drives predominately NHEJ [58]. However, 53BP1 also participates in reducing S-phase DNA damage and mitigating replication stress [59]. A maximal DDR function requires MDC-mediated recruitment of 53BP1 to chromatin through phospho-Ser139 H2AX [60]. However, when MDC1 interacts with chromatin in the absence of DNA damage, it does so in a γH2AX-independent manner through a proline-serine-threonine repeat (PST) domain that contributes to 53BP1 chromatin retention [60, 61]. We carried out experiments in the absence, versus the addition, of exogenous DNA damaging agents and found an MDM2-dependent interaction of 53BP1 interacting with MDC1 (Fig. 5). Damaged DNA creates chromatin territories by MDC1 alternatively regulating the DNA damage sensor MRN and Rad51 to promote HR, or 53BP1 to interact with RIF1 and the Shieldin complex to protect DNA ends from nucleolytic attack by MRN and subsequently promote NHEJ repair [57].

MDM2 is known to negatively regulate wtp53 function. However in the context of mtp53, the data presented here suggest a role reversal, in which mtp53 regulates MDM2. Using the p53–MDM2 interaction disruptors N3a and ALRN-6924, we phenocopied the loss of the 53BP1–MDC1 PLA interaction displayed by MDM2-depleted T47D cells (Figs 5 and 6). This suggests the MDM2–mtp53 complex mediates the 53BP1–MDM2 interaction. Additionally, we provide evidence for MDM2-mediated negative regulation of PARP1 activity in the context of mtp53, as loss of MDM2 in T47D cells resulted in enhanced cellular protein PARylation (Fig. 7). Interestingly, in the context of either p53–MDM2 disruptor the PARP1 protein levels within T47D cells were stable (Supplementary Fig. S4C). In stark contrast MCF7 cells treated with N3a or ALRN-6924 underwent apparent MDM2-promoted ubiquitin mediated proteolysis of PARP [31]; Supplementary Fig. S4C). Thus, regulation of MDM2 activity may enhance mtp53 properties that contribute to GOF. Indeed, we speculate that the mtp53-MDM2 complex is the active form of mtp53 that mediates DNA replication, DNA repair, and perhaps other mtp53 GOF activities. The MDC1–53BP1 interaction promoted by MDM2 with mtp53 described here could be viewed as a mtp53 GOF activity that bypasses the need for ATM activation. This is particularly interesting because ATM is one enzyme responsible for phosphorylating 53BP1 at Ser25 and Ser1778 [62, 63]. The fact that wtp53 uses its C-terminus to facilitate the recruitment 53BP1 for DNA repair may explain a mtp53 coopted function [64]. This interpretation of a coopted mtp53 GOF activity is consistent with that proposed for the mtp53–TopBP1 interaction that drives DNA replication [25]. In this mtp53 GOF pathway, mtp53 abrogates the requirement for both PI3 kinase and Cyclin/Cdk activity, which promotes the TopBP1–Treslin interaction and replisome assembly [25]. Recently, the active form of Treslin in DNA replication has been shown to be a complex with MTBP, a protein identified as an MDM2 binding protein. In light of this discovery, a role for MDM2 in the mtp53–TopBP1–Treslin pathway merits investigation.

Mutant p53 participates in cancer cell DNA replication, promotes tumorigenesis, and activates metastasis [22, 33, 65, 66]. Our previous work has focused on the interaction of mtp53 with PARP and PARylated chromatin [22, 23, 54, 55]. MDM2 is known for regulating DNA replication, chromatin, and DNA repair by down-regulating PARP and wtp53 [31, 67]. However, the ability of mtp53 to work with MDM2 to influence DNA replication and repair is addressed herein for the first time. As such, upon discovering the ability of MDM2 to recruit MDC1 and 53BP1 to chromatin we became particularly interested in how MDM2 regulated mtp53-based PARP1 functionality. In conditions of cancer cells with mtp53 (using two different breast cancer cell lines) we found that reduction of MDM2 did not increase PARP protein level but did result in more PARylation of chromatin (Fig. 7). This suggests that in the absence of MDM2, when MDC1 and 53BP1 levels on chromatin are decreased, the PARP DNA repair pathway is activated. This contrasts with what is seen in cells with wtp53, which is that the inhibition of MDM2 interacting with wtp53 causes both an increase in wtp53 and MDM2 protein and a decrease in total PARP levels because the MDM2 then works on PARP as an E3 ubiquitin ligase promoting its degradation (see Supplementary Fig. S4C). The scenario in cancer cells for mtp53 and PARP is that the depletion of MDM2 does not cause either mtp53 or PARP levels to dramatically increase (see Figs 1–6). The observation that ATM limits MDM2-mediated degradation may explain high mtp53 levels (Fig. 6). However, enhanced MDM2 activity in the presence of ATMi does not result in PARP1 degradation (unpublished observations), indicating other mechanism(s) may be at play. These could involve GOF mtp53 protection of PARP from MDM2-mediated degradation while promoting alternative mechanisms for MDM2 to inhibit PARP enzymatic activity. Future experiments are required to elucidate the complex cross-talk between MDM2, mtp53, 53BP1, MDC1, and PARP in the context of cancer cell DNA replication stress. Together mtp53, MDM2, 53BP1, MDC1, and PARP proteins may coordinate to facilitate CPR, thus allowing cancer cells to survive with replication stress and chromosomal instability that results in an immunosuppressive tumor microenvironment [33].

## Supplementary Material

gkaf627_Supplemental_File

## Data Availability

Data are available via ProteomeXchange with identifier PXD061454. Raw data can be found on Zenodo: https://zenodo.org/uploads/13337033?token=eyJhbGciOiJI  UzUxMiJ9.eyJpZCI6IjBlOTk5MzMzLTNmZWItNGY1NC  04MzVjLTA3NWZjOGI5MDhkMiIsImRhdGEiOnt9LCJy  YW5kb20iOiJhMTliMGU2ZmFhYzBkZDJmMmFkYWI  yMGE4Njg2MWY4NyJ9.g10HVzuTiO8BOzZ8N4cSoi  hVUHsYpEMBtxs3l-LN76X4IfY4M4EUHfHUadIWfTB  bjnkAkAgLma7RabR9mLDMwg.
