# Supplementary material for: A cancer persistent DNA repair circuit driven by MDM2, MDM4 (MDMX), and mutant p53 for recruitment of MDC1 and 53BP1 on chromatin
Source: Nucleic Acids Res. 2025 Jul 8;53(13):gkaf627. doi: 10.1093/nar/gkaf627 (PMC12235508; doi:10.1093/nar/gkaf627)
Supplement: gkaf627_Supplemental_File [file gkaf627_supplemental_file.pdf]

NAR 53BP1-MDM2 Paper Supplemental Data

S1: siRNA mediated knockdown of 53BP1 in T47D decreases p53-53BP1, MDM2-53BP1, and MDC1-53BP1 PLA foci

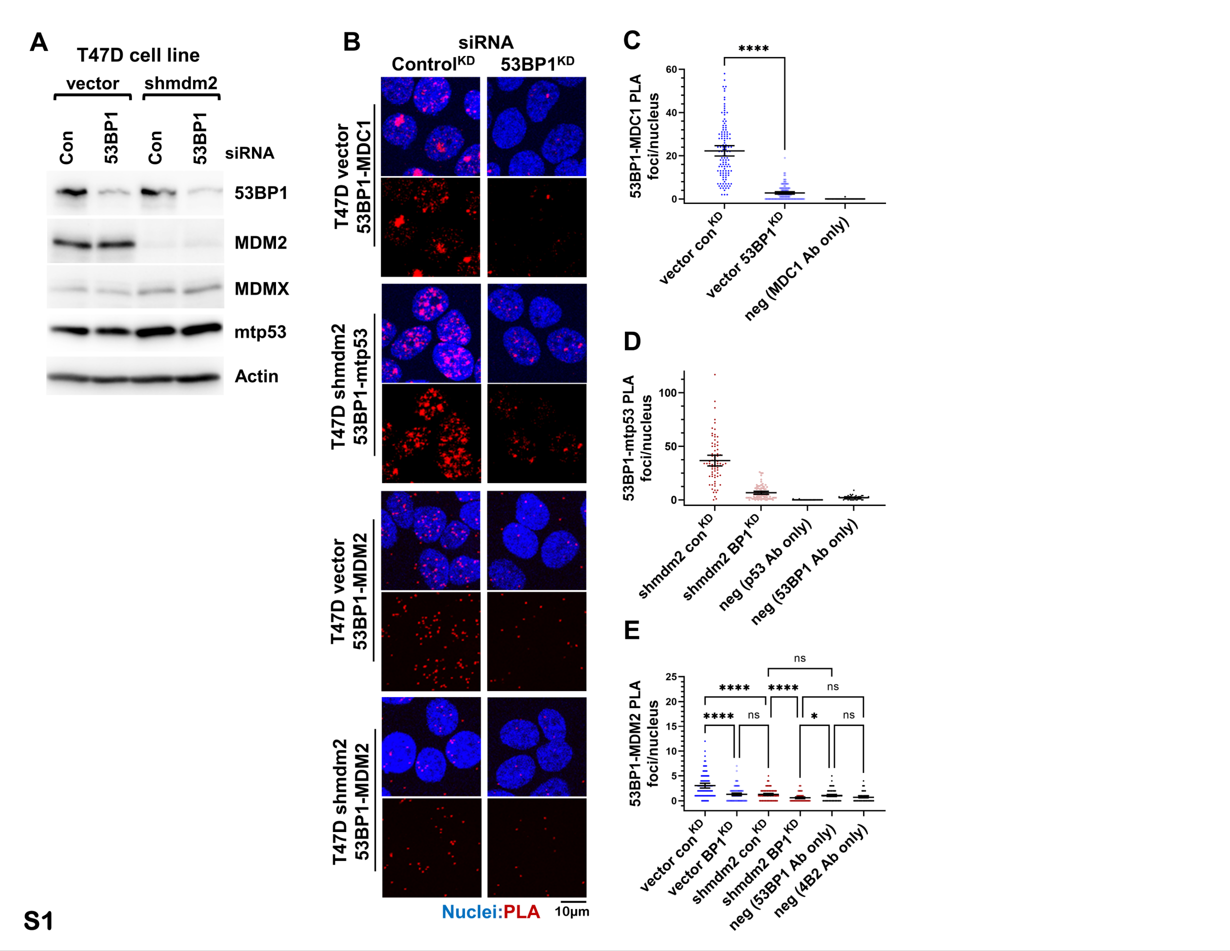

(A) Western blot analysis for the indicated proteins within extracts (10 μg) from T47D *vector* and *shmdm2* populations 72hr post-transfection with either control siRNA (Con) or 53BP1 siRNA (53BP1). **(B-E)** PLA analysis of MDC1-53BP1 (panel C), 53BP1-mtp53 (panel D), and MDM2-53BP1 (Panel E) in T47D *vector* and *shmdm2* populations 72hr post-transfection with either control siRNA (Con) or 53BP1 siRNA (53BP1). PLA analysis of the indicated proteins was measured using PLA rabbit anti-MDC1, anti-p53, PLA goat anti-53BP1, and mouse anti-MDM2 4B2 antibodies. Confocal images for ≥3 fields for each were acquired and the number of PLA foci/nucleus for each cell population was quantified. Representative data (images in Panel B and graphs in Panel C-E) with mean, 95% CI, and Kruskal-Wallis statistical significance test prepared as described in the materials and methods from the indicated number of cells in Panel: (C) *vector* con= 119, *vector* 53BP1= 139, neg MDC1 Ab only= 127; (D) *shmdm2* con= 75, *shmdm2* 53BP1= 80, neg p53 Ab only= 82, neg 53BP1 Ab only= 53; (E) *vector* con= 98,

vector 53BP1= 111, *shmdm2* con= 117, *shmdm2* 53BP1= 112, neg 53BP1 Ab only= 96, neg 4B2 Ab only= 103.

S2: *mtp53 T47D cell cycle progression is not halted by Nutlin 3a*

A

Fraction S/G2 population 24hr-post Nultin 3a treatment in T47D vector, shmdmx, and shmdm2 cells

	vector (n=289)	vector (+N3a) (n=227)	shmdmx (n=278)	shmdmx (+N3a) (n=283)	shmdm2 (n=249)	shmdm2 (+N3a) (n=245)
EdU-labeled Cyclin A+	76/289 (26.3%)	69/227 (30.4%)	94/278 (33.8%)	64/283 (22.6%)	76/249 (30.5%)	57/245 (23.3%)
EdU-negative Cyclin A+	21/289 (7.3%)	18/227 (7.9%)	11/278 (4.0%)	18/283 (6.4%)	16/249 (6.3%)	15/245 (6.1%)

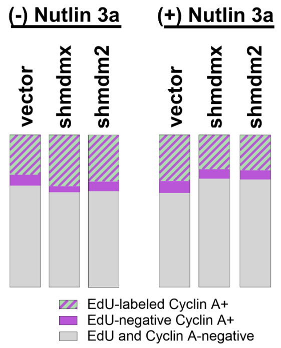

B

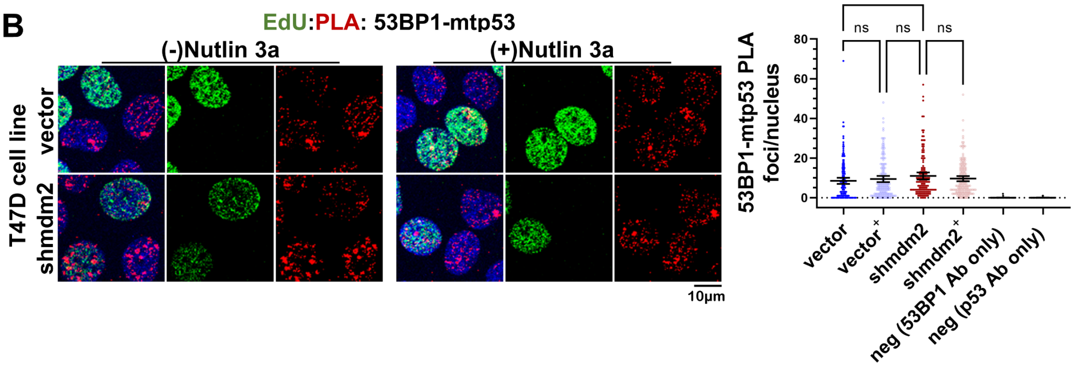

(A) Comparable S/G2 fraction within untreated and Nutlin 3a treated T47D cell populations. 24 hr post-treatment with either vehicle or 10μM Nutlin 3a, cells were labeled with EdU for 20 minutes and assayed for Cyclin A2 by immunofluorescence. Confocal images from at least 3 fields were acquired (presented in Fig. 4 Panel B) and the number of EdU+/Cyclin+ (S-phase) and EdU-/Cyclin A+ (G2/M) nuclei were quantified in each population; tabulated data on the left represented as bar graphs on the right. **(B)** p53- 53BP1 PLA interactions are refractory to Nutlin 3a. T47D *vector* and *shmdm2* cells were treated with vehicle or Nutlin 3a (labeled "+") for 24 hr, labeled with EdU for 20 min, and then assayed for 53BP1-p53 PLA foci. The number of PLA foci/nucleus from each cell population was determined from confocal images (representative images presented on the left) and data representation (right graph) with mean, 95% CI, and Kruskal-Wallis statistical significance test was prepared from the indicated number of cells: *vector*= 159; *vector*$^{+}$= 155; *shmdm2*= 147; *shmdm2*$^{+}$= 167; neg 53BP1 Ab only= 184; neg p53 Ab only= 179.

S3: MDM2 required for stable MDC1-53BP1, but not 53BP1-DNA, PLA interactions

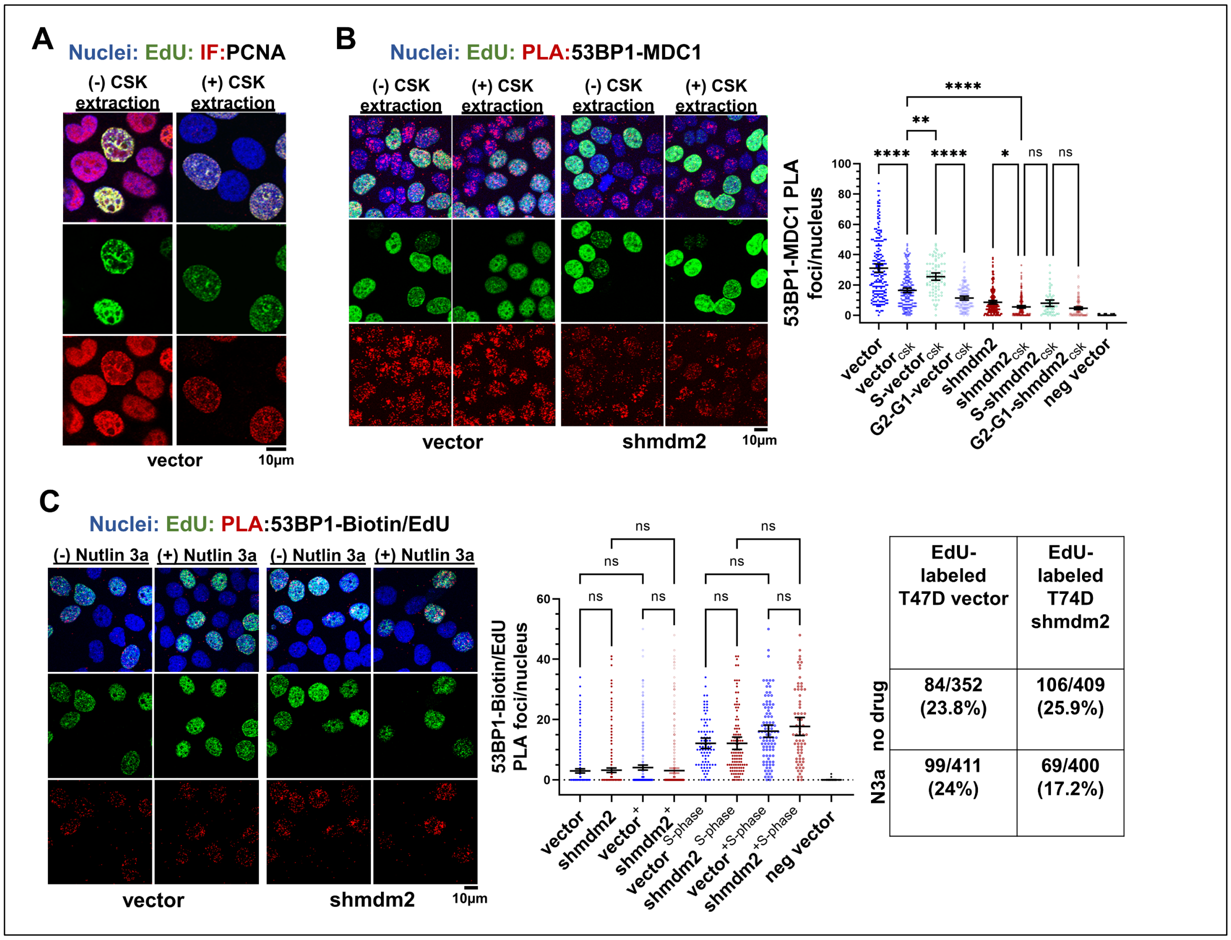

	EdU-labeled T47D vector	EdU-labeled T74D shmdm2
no drug	84/352 (23.8%)	106/409 (25.9%)
N3a	99/411 (24%)	69/400 (17.2%)

(A-B) 53BP1-MDC1 PLA CSK-extraction resistant foci requires MDM2. Post labeling with EdU, T47D *vector* and *shmdm2* were incubated with CSK extraction buffer for 7 min at 4°C before immunofluorescence for PCNA (Panel A) or 53BP1-MDC1 PLA foci (Panel B). The number of PLA foci/nucleus from each cell population was determined from confocal images and graphed as both PLA foci/total number of nuclei and binned based on EdU (+/-) status. Representative data with mean, 95% CI, and Kruskal-Wallis statistical significance test prepared as described in the materials and methods from the indicated number of cells: *vector* (-) CSK= 201; *vector* (+) CSK (total= 223, S-phase= 81, G1-G2= 142); *shmdm2* (-) CSK= 199; *shmdm2* (+) CSK (total= 200, S-phase= 53, G1-G2= 147). **(C)** 53BP1-DNA PLA foci form independent of MDM2. T47D *vector* and *shmdm2* cells were treated with vehicle or Nutlin 3a (labeled "+") for 24 hr, labeled with EdU for 20 min, and then assayed for 53BP1-DNA PLA foci using PLA goat anti-53BP1 Ab and mouse anti-Biotin Ab (a mixture of 5:1 Biotin-N_3 and AlexaFluor-N_3 was coupled to EdU using click chemistry). The number of PLA foci/nucleus from each cell population was determined from confocal images and graphed as both PLA foci/total number of nuclei and binned based on EdU status. Representative data with mean,

95% CI, and Kruskal-Wallis statistical significance test prepared as described in the materials and methods from the indicated number of cells: vehicle-treated [*vector* (total= 352, S-phase= 84); *shmdm2* (total= 409, S-phase= 106)] and Nutlin 3a-treated [*vector* (total= 411, S-phase= 99); *shmdm2* (total= 400, S-phase= 69)].

S4: *MDM2-p53 disruptors block cell cycle progression and reduce MDC1-53BP1 PLA foci in MCF7 cells*

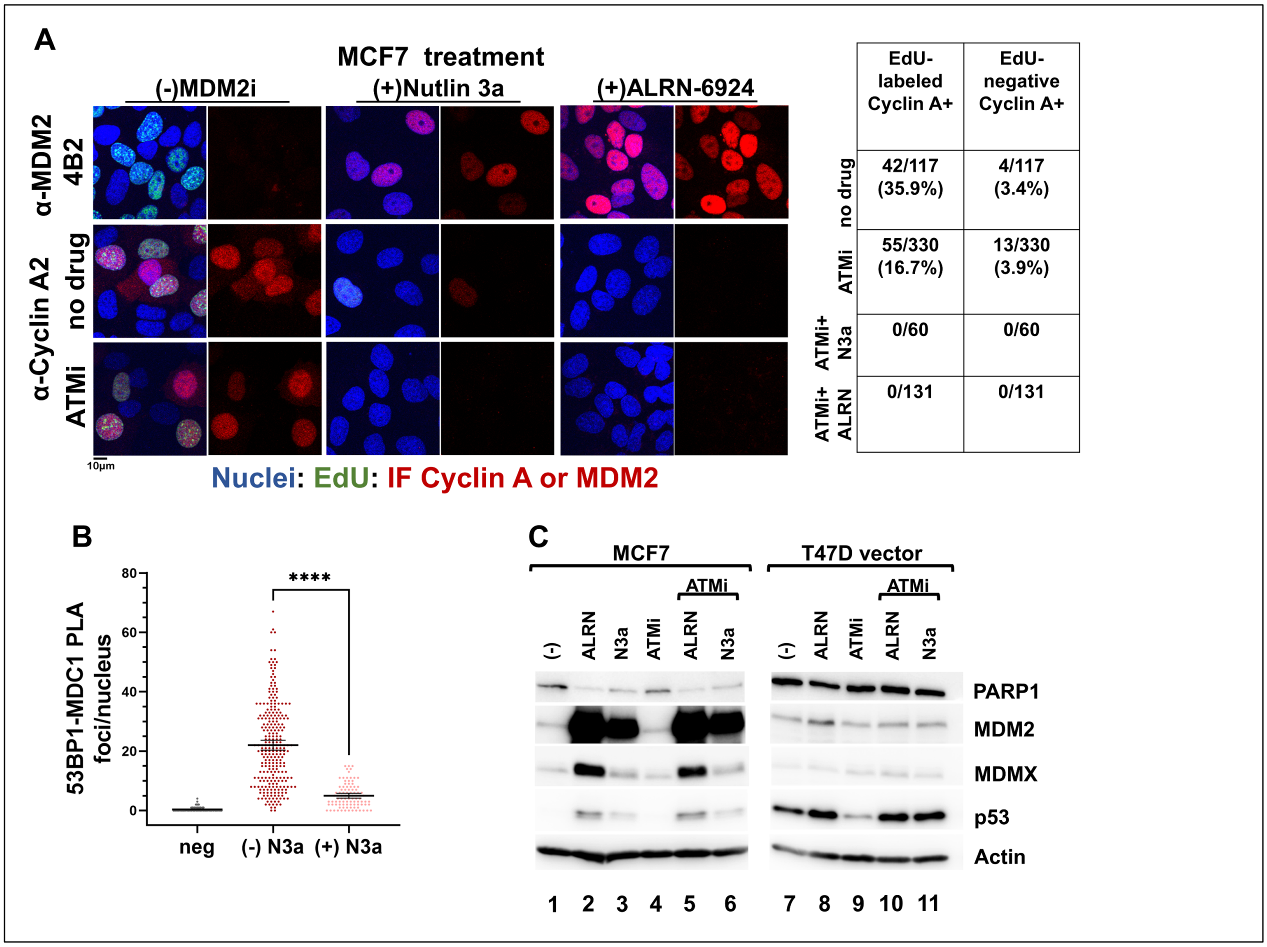

(A) 24hr post treatment with either vehicle,10 µM Nutlin 3a, 10 µM ALRN-6924 (MDM2/X dual inhibitor), 10 µM KU-55933 (ATMi; ATM inhibitor) or MDM2i+ATMi, MCF7 cell populations were labeled with EdU for 20 minutes and assayed for Cyclin A2 and MDM2 (positive control for drug bioactivity) by immunofluorescence. Confocal images from several fields were acquired and the number of EdU+/CyclinA+ (S-phase) and EdU-/Cyclin A+ (G2/M) nuclei were quantified. **(B)** Reduced MDC1-53BP1 PLA foci in MCF7 24 hr post-treatment with Nutlin 3a. PLA analysis was performed on MCF7 cell populations in the absence or 24hr post Nutlin 3a treatment (labeled "+"), and the number of PLA foci/nucleus determined from the following cell populations: neg MDC1

Ab only= 133; MDC1-53BP1= 265; MDC1-53BP1^{+}= 96. **(C)** Inhibition of ATM promotes MDM2 activity. Western blot analysis for the indicated proteins within extracts (10 µg) from MCF7 and T47D populations treated for 24hr with either vehicle, 10 µM Nutlin 3a, 10 µM ALRN-6924 (MDM2/X dual inhibitor), 10 µM KU-55933 (ATMi; ATM inhibitor) or a combination both MDMi+ATMi.

S5: *MDM2 reduces poly-ADP-Ribose (PAR) levels of chromatin-bound proteins in MDA-MB-231*

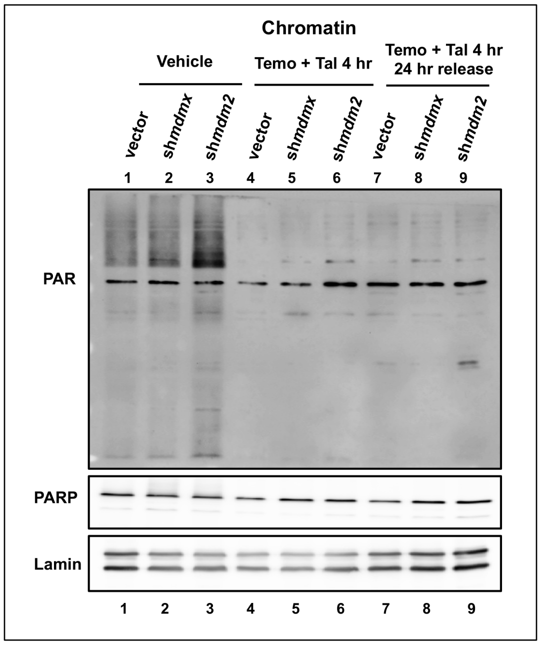

MDA-MB-231.mlp (vector control) and MDA-MB-231.mlp.sh*mdm2* and sh*mdmx* cell lines were plated and treated with or without temozolomide or talazoparib for 4 hr. In parallel, cells were treated with temozolomide or talazoparib for 4 hr followed by a wash with 1x PBS and allowed a 24 hr recovery period post temo/tal treatment in 1x DMEM media supplemented with 10% FBS and 5% Penn-strep. Cells were harvested and lysed according to the chromatin fractionation protocol. Protein levels for PAR and PARP, MDM2/X, Actin, and Lamin were semi-quantified by immunoblot analysis.
